# Machine learning and bioinformatics analysis revealed classification and potential treatment strategy in stage 3–4 NSCLC patients

**DOI:** 10.1186/s12920-022-01184-1

**Published:** 2022-02-22

**Authors:** Chang Li, Chen Tian, Yulan Zeng, Jinyan Liang, Qifan Yang, Feifei Gu, Yue Hu, Li Liu

**Affiliations:** 1 Cancer Center, Union Hospital, Tongji Medical College, Huazhong University of Science and Technology, Wuhan, 430022 China; 2 Department of Ultrasound, Union Hospital, Tongji Medical College, Huazhong University of Science and Technology, Wuhan, 430022 China

**Keywords:** Immunophenotypes, Machine learning, Signature, Multiomics, Cancer immunotherapy, Drug sensitivity, Treatment strategy

## Abstract

**Background:**

Precision medicine has increased the accuracy of cancer diagnosis and treatment, especially in the era of cancer immunotherapy. Despite recent advances in cancer immunotherapy, the overall survival rate of advanced NSCLC patients remains low. A better classification in advanced NSCLC is important for developing more effective treatments.

**Method:**

The calculation of abundances of tumor-infiltrating immune cells (TIICs) was conducted using Cell-type Identification By Estimating Relative Subsets Of RNA Transcripts (CIBERSORT), xCell (xCELL), Tumor IMmune Estimation Resource (TIMER), Estimate the Proportion of Immune and Cancer cells (EPIC), and Microenvironment Cell Populations-counter (MCP-counter). K-means clustering was used to classify patients, and four machine learning methods (SVM, Randomforest, Adaboost, Xgboost) were used to build the classifiers. Multi-omics datasets (including transcriptomics, DNA methylation, copy number alterations, miRNA profile) and ICI immunotherapy treatment cohorts were obtained from various databases. The drug sensitivity data were derived from PRISM and CTRP databases.

**Results:**

In this study, patients with stage 3–4 NSCLC were divided into three clusters according to the abundance of TIICs, and we established classifiers to distinguish these clusters based on different machine learning algorithms (including SVM, RF, Xgboost, and Adaboost). Patients in cluster-2 were found to have a survival advantage and might have a favorable response to immunotherapy. We then constructed an immune-related Poor Prognosis Signature which could successfully predict the advanced NSCLC patient survival, and through epigenetic analysis, we found 3 key molecules (HSPA8, CREB1, RAP1A) which might serve as potential therapeutic targets in cluster-1. In the end, after screening of drug sensitivity data derived from CTRP and PRISM databases, we identified several compounds which might serve as medication for different clusters.

**Conclusions:**

Our study has not only depicted the landscape of different clusters of stage 3–4 NSCLC but presented a treatment strategy for patients with advanced NSCLC.

**Supplementary Information:**

The online version contains supplementary material available at 10.1186/s12920-022-01184-1.

## Background

Non-small cell lung cancer (NSCLC) is the most common type of lung cancer, which is the leading cause of cancer-related death worldwide [1]. The majority of NSCLC cases are often first diagnosed at an advanced stage when curative treatment is less effective [2]. The overall survival of non-small cell lung cancer patients is dissatisfied, and the high rate of invasion and metastasis are major problems [3, 4]. During the past two decades, ICIs (immune checkpoint inhibitors), including monoclonal antibodies targeting programmed death 1 (PD-1) and cytotoxic T-lymphocyte antigen-4 (CTLA-4) and combination immunotherapy, have begun to alter clinical treatment strategy in multiple cancers, especially in NSCLC [5]. Response to immune checkpoint inhibitors treatment is associated with multiple factors, such as tumor mutation burden (TMB), microsatellite instability (MSI), and PDL1 expression [6]. The efficacy of cancer immunotherapy also depends on the tumor stage [7]. Despite recent advances in cancer immunotherapy, the 5-year overall survival rate of advanced NSCLC patients remains low [8, 9]. Understanding the tumor microenvironment and heterogeneity in advanced NSCLC is important for developing more effective treatments [10].

The tumor microenvironment is a highly complex ecosystem. We assumed that the heterogeneity of advanced NSCLC could be distinguished based on the major cellular components of TME. The development of next-generation sequencing and public database have made it possible to explore novel treatment in multiple cancers [11, 12]. To obtain insight into the tumor microenvironment, many computational methodologies have been developed (including CIBERSORT, TIMER algorithms). For example, the CIBERSORT algorithm, which was termed as cell deconvolution approach, has been developed to infer lymphocytes and other immune cells proportions from bulk transcriptome data. These computational approaches help researchers identify specific cell types, and have been widely used in cancer studies [13]. Multi-omics analysis has deepened our understanding of the biological basis of cancer and precise survival prediction of patients, which is in line with the concept of precision medicine [14]. In this study, we attempted to classify advanced NSCLC patients, depict their characteristics, and identify novel therapeutic molecular targets or potential drugs for different clusters of patients.

## Method

### Data pre-processing

The bulk RNA-seq TCGA-LUAD and TCGA-LUSC data for NSCLC were downloaded as HTSeq-FPKM files from UCSC Xena (https://xenabrowser.net/datapages/). The corresponding clinical information including follow-up data was also collected from UCSC Xena database. TCGA-LUAD and TCGA-LUSC microRNAs data were derived from TCGA data portal (https://portal.gdc.cancer.gov/). The expression profiles of TCGA-LUAD and TCGA-LUSC were pre-processed by the following steps: 1) Removing samples without follow-up information; 2) Preserving stage 3 or stage 4 samples; 3) The expression profile (FPKM values) was transformed into TPMs; 4) Preserving the genes of log2 (TPM + 1) > 0. From this, 195 advanced NSCLC samples from TCGA cohort were sorted out for further analysis.

Additional cohorts of NSCLC patients were derived from Gene Expression Omnibus (http://www.ncbi.nlm.nih.gov/geo/, platform Illumina GPL6884 (n = 116): GSE41271 and GSE42127; platform Affymetrix GPL570 (n = 45): GSE29013 and GSE37745). The potential batch effects (Specifically, between GSE41271 and GSE42127, and between GSE29013 and GSE37745) were eliminated using ComBat function (“SVA” package in R) [15]. The detailed information of the studying cohorts was summarized in Additional file 2: Table S1-S2 (Combined affy cohort, GSE29013 + GSE37745, N = 45, Additional file 2: Table S1; Combined illumina cohort, GSE41271 + GSE42127, N = 116, Additional file 2: Table S2).

Transcriptomic and the corresponding clinical information of patients with urothelial cancer treated with atezolizumab (anti-PD-L1) was downloaded from Imvigor210 (http://research-pub.gene.com/IMvigor210CoreBiologies/), clinical endpoints including complete or partial response (CR or PR), stable disease (SD), and the progressive disease (PD). Another two transcriptomic datasets from patients with NSCLC treated with PD-1 blockade were downloaded from GSE126044 and GSE135222 (Two anti-PD1 treatment cohorts, N = 27, N = 16, Additional file 2: Table S3; Imvigor cohort, N = 348, Additional file 2: Table S4).

### Estimation of the immunological characteristics of advanced NSCLC

The abundance of LM22 (22 immune cell types) was calculated using CIBERSORT algorithm [16] (model = relative, permutation = 1000, disable quantile normalization = True, https://cibersort.stanford.edu/). To avoid calculation errors, we comprehensively calculated the abundance of immune cells using another four algorithms: TIMER, xCell, EPIC, MCP-counter [17–20]. The immune score, stromal score, and ESTIMATE score for each sample were calculated by applying “ESTIMATE” function in R [21]. In addition, Eighteen immune-related therapeutic signatures were collected from the Jiao Hu et al. study [22], and 23 immune-related gene sets were collected from MSigDB database and previous publications [23]. Effector genes of immune cells were identified from previous publications [24]. To predict clinical outcome and response to ICI therapy among different sub-clusters, four response signatures reported previously [25–27] were calculated using ssGSEA. We used these TIICs abundances or genesets to depict the immune-related parameters of the studying cohorts.

### Identification of clusters based on consensus clustering

Unsupervised clustering methods were performed (K-means, “ConsensusClusterPlus” package in R) to determine sub-clusters (applied in TCGA cohort and two independent external validation cohorts, including affy-combined cohort and illumina-combined cohort) based on LM22 [28, 29]. This procedure was repeated 1000 times to ensure classification stability.

### Construction of poor prognosis signature

The NSCLC samples in TCGA cohort were randomly assigned into the training/validation cohort (6:4). A total of 2720 immune-related genes were collected from InnateDB (https://www.innatedb.com) and Immport (https://www.immport.org/). The immunological characteristics mentioned above (Estimation of the immunological characteristics of advanced NSCLC) were calculated separately using respective methods (The abundance of immune cells, calculated by TIMER, xCell, EPIC, MCP-counter algorithm; The immune score, stromal score, and ESTIMATE score, calculated using the ESTIMATE algorithm; The enrichment of immune-related signatures, calculated using ssGSEA). All variables were merged into a feature matrix, and feature engineering was performed to filter survival-unrelated and cluster 2-irrelevant variables. Then all features were standardized across all samples (features were standardized using Z-score normalization), and LASSO-penalized regression was conducted [30] to further reduce the number of features (“glmnet” package in R). Among features identified in LASSO analysis, multivariate cox regression analysis was conducted, and Poor Prognosis Signature (PPS) was constructed by applying the regression coefficients.

### Construction of classifiers to distinguish different subclusters based on machine learning

To simplify and find the best approach to distinguish different advanced NSCLC sub-clusters (determined by K-M clustering), four different algorithms, including SVM (Support Vector Machine) [31], RF (Randomforest) [32], Xgboost (eXtreme Gradient Boosting) [33, 34] and Adaboost (Adaptive boosting) [35], were recruited to build up the classifier. We attempted to find the best parameters of different algorithms. Specifically, for SVM, cross-validation and grid search were applied to find out the best model parameters (cost = 8 and gamma = 0.00391); for RF, we selected mtry = 18 and ntree = 800 as the best parameters, and random forest method has an internal validation method; for Xgboost and Adaboost, we extracted 80% samples randomly to assess the classifier and this procedure was repeated 1000 times. We built up the classifier in the training cohort and compared their performance in the validation cohort. For every algorithm, the performance measures included accuracy, precision, recall, F1 score, and AUC.

### Drug sensitivity

CTRP (Cancer Therapeutics Response Portal) and PRISM (Profiling Relative Inhibition Simultaneously in Mixture), which contains the sensitivity data for more than 1000 compounds, were used to generate drug sensitivity data [36, 37]. Both databases provide AUC values as a measure of drug sensitivity, and higher AUC values indicate decreased sensitivity to specific compounds. Any compound or drug with more than 20% missing values was excluded before inferential analysis [14].

### Calculation of TMB

Non-synonymous mutations were defined as "Frame_Shift_Del", "Frame_Shift_Ins", "Missense_Mutation", "Nonsense_Mutation", "Splice_Site", "In_Frame_Del", "In_Frame_Ins", "Translation_Start_Site", "Nonstop_Mutation", and the exome size was defined as 38 Mb [38]. TMB was calculated by this formula:

TMB = (Non-synonymous mutations)/ (exome size).

### Copy number variation, DNA Methylation, and miRNA analysis

The TCGA CNV data (Masked copy number Segment hg38) was derived from TCGA database. Values of segment mean bigger than 0.1 were defined as gain and less than -0.1 as a loss. All CNV data was analyzed using GISTIC 2.0 [39].

Methylation data using Illumina Human Methylation 450 k was obtained from UCSC Xena browser. R package “Champ” was utilized for normalization and “limma” for the identification of differentially methylated probes [40].

R package “edgeR” was utilized to determine differentially expressed miRNA. MiRNA-DEG links were predicted by different miRNA databases (miRDB, mirTarbase, Targetscan, predictions in at least two databases were defined as positive predictions) [41–43].

### Gene set enrichment analysis and differentially expressed gene analysis

To determine which pathways or biological functions differ between different sub-clusters, GSEA (version: 4.0) was performed. *C5.go.bp.v7.2.symbols.gmt*, *c2.cp.kegg.v7.2.symbols.gmt* and *h.all.v7.2.symbols.gmt* set as reference gene sets. Differentially expressed genes were identified using “limma” package in R, and the thresholds were set as |log2-fold change |> 1.0 and F*df* < 0.05.

### protein–protein interaction network

The PPI network of the key proteins identified in the multi-omic analysis was constructed using the STRING database (https://string-db.org/), and parameters were set to default values [44].

### Bioinformatic analysis

The bioinformatic analysis involved in our study included: (a). Preprocessing and analysis of the transcriptome data, mutation data, and copy number alteration data. (b). Calculation of immune cell abundance using CIBERSORT, TIMER, xCell, EPIC, MCP-counter, and ESTIMATE algorithms. (c). GSEA and ssGSEA (single sample GSEA) were used to calculate an enrichment level of certain signatures in different groups or samples. (d). miRDB, mirTarbase, and TargetScan databases were used for the miRNA target prediction. (e). Classified patients into different groups using unsupervised KM clustering. (f). Construction of PPS model using LASSO-COX analysis. (g). Construction of the classifier using different MLs (RF, XGBoost, Adaboost, and SVM) and DL (NNet). (h). Drug sensitivity data (derived from CTRP and PRISM) analysis using ridge regression. (i). Protein–protein interaction analysis using STRING.

### Statistical analysis

Normality was calculated via the Shapiro–Wilk normality test. Wilcoxon test and Kruskal–Wallis test were utilized to analyze the ordered categorical variables. Student’s t- or chi-square test was used to compare continuous or discrete variables. Statistical analysis was two-sided, and *P* < 0.05 was considered to be statistically significant. To avoid false positives in multiple tests as much as possible, we performed the false discovery rate correction. All these analyses were conducted through R software.

## Result

### The landscape of advanced NSCLC TME

CIBERSORT algorithm was performed to quantify the abundance of LM22 in TCGA advanced NSCLC samples (stage 3 and stage 4 TCGA-LUSC, TCGA-LUAD, N = 195, Table 1). To avoid the calculation errors due to marker gene sets of tumor-infiltrating immune cells (TIICs), we estimated the abundance of immune cells using four other algorithms (TIMER, xCell, EPIC, MCP-counter), and compared the correlations among them. Five TIICs overlapping in different algorithms, including CD8 + T cell, M2.macrophage, M1.macrophage, Neutrophil, Dendritic cell, have shown a high degree of similarity with the results calculated by CIBERSORT (Additional file 1: Figure S1). E.g., the enrichment level of CD8 + T cell quantified by the four independent algorithms was in line with the previous CIBERSORT results (Spearman correlation, TIMER: 0.67, xCell: 0.80, EPIC: 0.71, MCP-counter: 0.65, Additional file 1: Figure S1, Additional file 2: Table S5), which demonstrated the stability of calculation. Unsupervised clustering (K-means) was performed to classify the advanced NSCLC into different sub-clusters based on TIICs level of the 195 tumor samples. We assessed the clustering parameters (Additional file 1: Figure S2 A-B) and the optimal cluster number was set as three. Samples from the TCGA cohort were then assigned to three separate clusters (cluster 1, n = 79; cluster 2, n = 61; cluster 3, n = 55). The clinical information was shown in Supplementary Material (Additional file 2: Table S6).

**Table 1:** Clinical information of patients in stage 3–4 TCGA-NSCLC cohort

Characteristics (N = 195)	No. cases
*Age*	
age < = 65	89
age > 65	105
NA	1
*Pathologic_M*	
M0	131
M1	22
M1a	3
M1b	6
NA	33
*Pathologic_N*	
N0	30
N1	46
N2	107
N3	7
NA	5
*Pathologic_T*	
T1	12
T1a	5
T1b	3
T2	65
T2a	15
T2b	6
T3	47
T4	39
NA	3
*Gender*	
female	77
male	118
*Stage*	
Stage 3	3
Stage 3a	132
stage 3b	28
stage iv	32
*Type*	
LUAD	105
LUSC	90

**Fig. 1:**
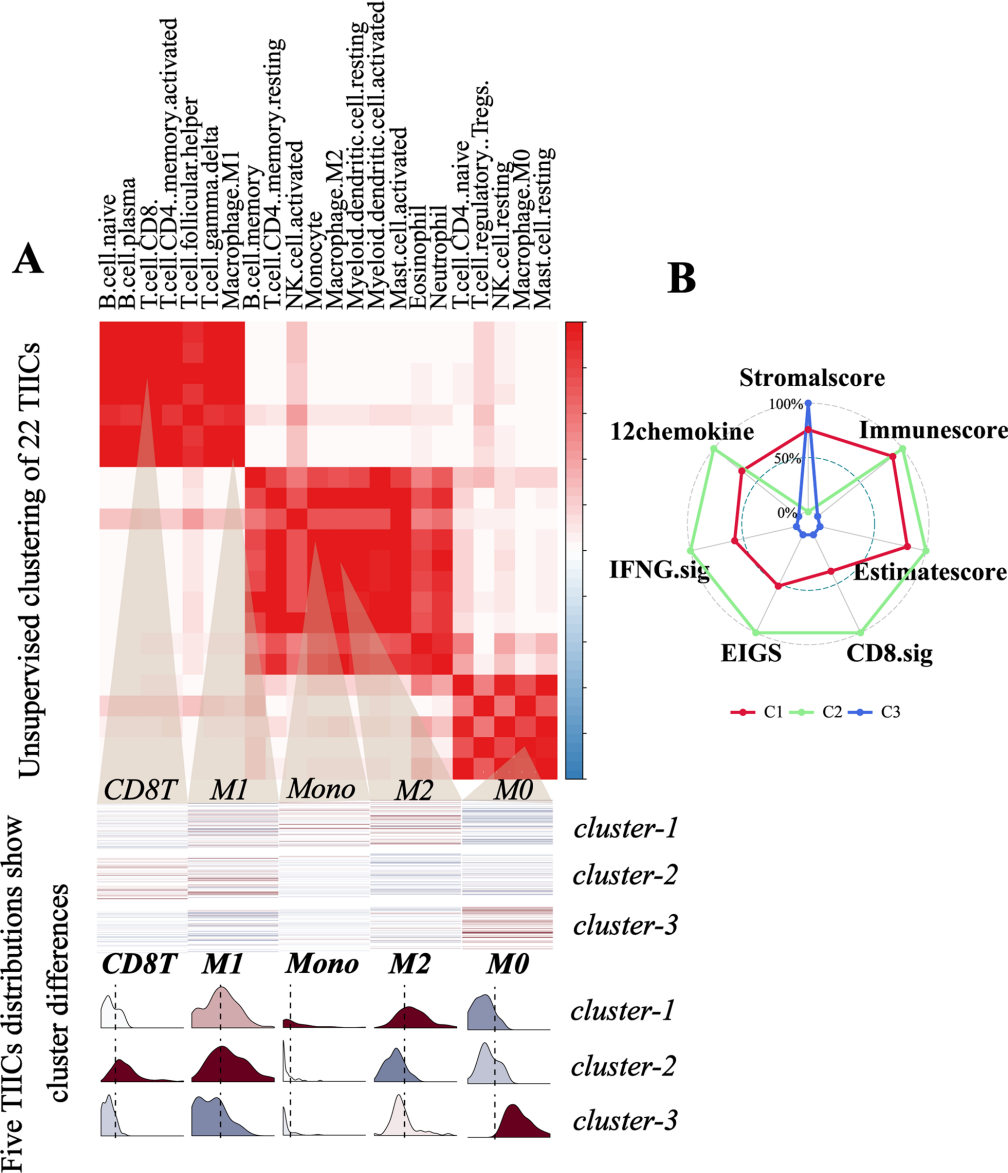
Unsupervised clustering of TIICS in stage 3–4 NSCLC. **A**. Top: Consensus clustering of the pairwise correlation of TIICs. Three modules associations were indicated in the heatmap. Middle: Five representative immune cells (T cells CD8, M1.Macrophage, Monocyte, M2.Macrophage, M0.Macrophage) from each module, with heatmap indicating the abundance (Dark colour represents high expression level, while the light colour represents the low expression level). Bottom: Distribution of the five selected TIICs within the three clusters (row), with dashed line indicating the median. **B** Radar graph indicates the ESTIMATE scores and four immunotherapy-related signature scores in three clusters. Line color represents the three clusters: Red for cluster-1, green for cluster-2, and blue for cluster-3

**Fig. 2:**
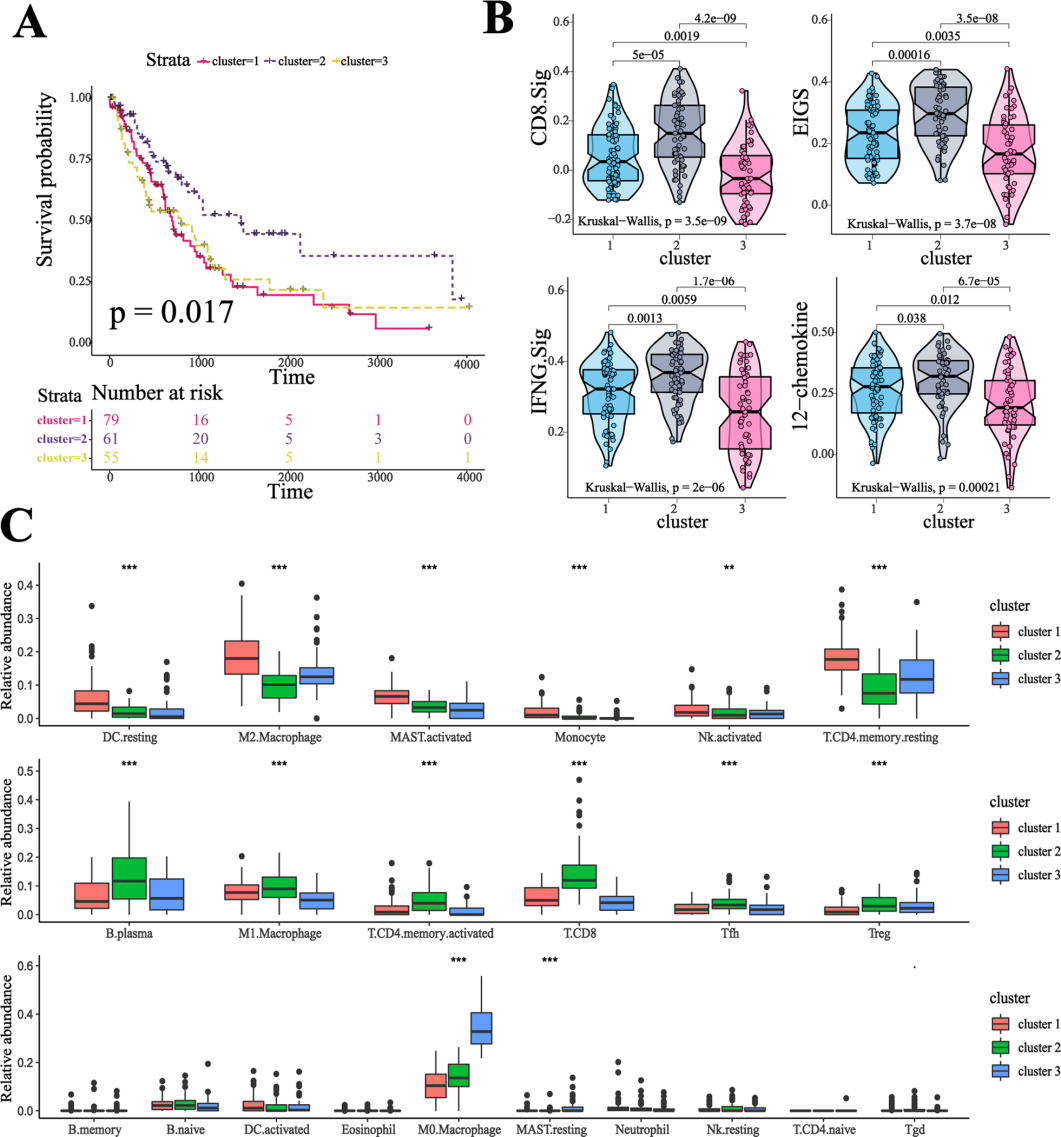
TME characteristics in each clusters. **A** Kaplan–Meier curve displaysdifferences of overall survival among three clusters. Log-rank statistic was conducted to test statistical significance. **B** Comparison of GSVA score of CD.Sig, IFNG.sig, EIGS, 12-chemokine signature among different clusters. Kruskal–Wallis statistic was conducted to test statistical significance. **C** 22 TIICs abundance among three clusters were shown in the box plot. ***, *P* < 0.0001; **, *P* < 0.001; *, 0.001 < *P* < 0.01

Cluster analysis revealed distinct immune infiltration patterns among these three clusters (Fig. 1A, Additional file 1: Figure S2C): cluster-1 was characterized by increases in the infiltration of resting DCs, M2.marcrophages, activated mast cells, monocytes, activated NKs, and resting CD4 + T memory cells; cluster-2 showed an evident increase in the infiltration of plasma, M1.macrophages, activated CD4 + T memory cells, CD8 + T cells, T follicular helper cells, and Tregs; cluster-3 exhibited a high infiltration of M0.macrophages and resting mast cells and exhibited decreases in other TIICs. The significant difference of TIICs infiltration in these three clusters was confirmed by Kruskal–Wallis tests (Fig. 2C). To investigate the association between TME phenotypes and clinical characteristics, clinical factors, including age, gender, tumor stage, lymph node metastasis, and distant metastasis, were analyzed. However, there was no significant difference in these clinical characteristics among the three clusters (Additional file 2: Table S6).

To unravel the biological differences among these clusters, selected chemokine and cytokine mRNA expression in the 195 samples were analyzed. Grossly, immune-activated-related molecules (CD8A, CXCL10, CXCL9, GZMA, GZMB, IFNG, PRF1, TBX2, and TNF) were relatively higher in cluster-2 compared to the other clusters; cluster-3 was associated with relatively low expression of immune-checkpoint-related molecules (CD274, CTLA4, HAVCR2, IDO1, LAG3, PDCD1, and PDCD1LG2), whereas expression of TGFβ/EMT-pathway-related molecules (ACTA2, CLDN3, COL4A1, SMAD9, TGFBR2, TWIST1, VIM, and ZEB1) were high (Additional file 1: Figure S3A-B). Then, we referred to a database of co-inhibitory, co-stimulatory, and MHC-related molecules to better compare these immunomodulators among these three clusters. Overall, the result showed that cluster-2 had a higher expression of co-inhibitors and co-stimulating molecules than the other clusters, while MHC-related molecules showed no significant difference among these clusters (Additional file 1: Figure S3C-D). In addition, cluster-2 was associated with higher expression of effector genes of CD8 + T cells as compared to cluster-1 and cluster-3 (Additional file 1: Figure S3E-F). These results indicated that cluster-2 tended to be an inflammatory phenotype, which indicated that patients classified into cluster-2 might have a better clinical outcome. Kaplan–Meier curve indicated that cluster-2 had an overall survival advantage (log-rank test, p = 0.017, Fig. 2A). ESTIMATE score and immune score were higher in cluster-2 (Fig. 1B). In addition, the GSVA score of four immunotherapy-related signatures was significantly higher in cluster-2 as compared to the other clusters (Kruskal–Wallis tests, CD8.sig, IFNG.sig, EIGS, 12-chemokines.sig, all p value < 0.001), which indicated that patients in cluster-2 might have a better response to ICI (immune checkpoint inhibitors) therapy (Fig. 2B).

### Construction of the poor prognosis-associated signature

**Fig. 3:**
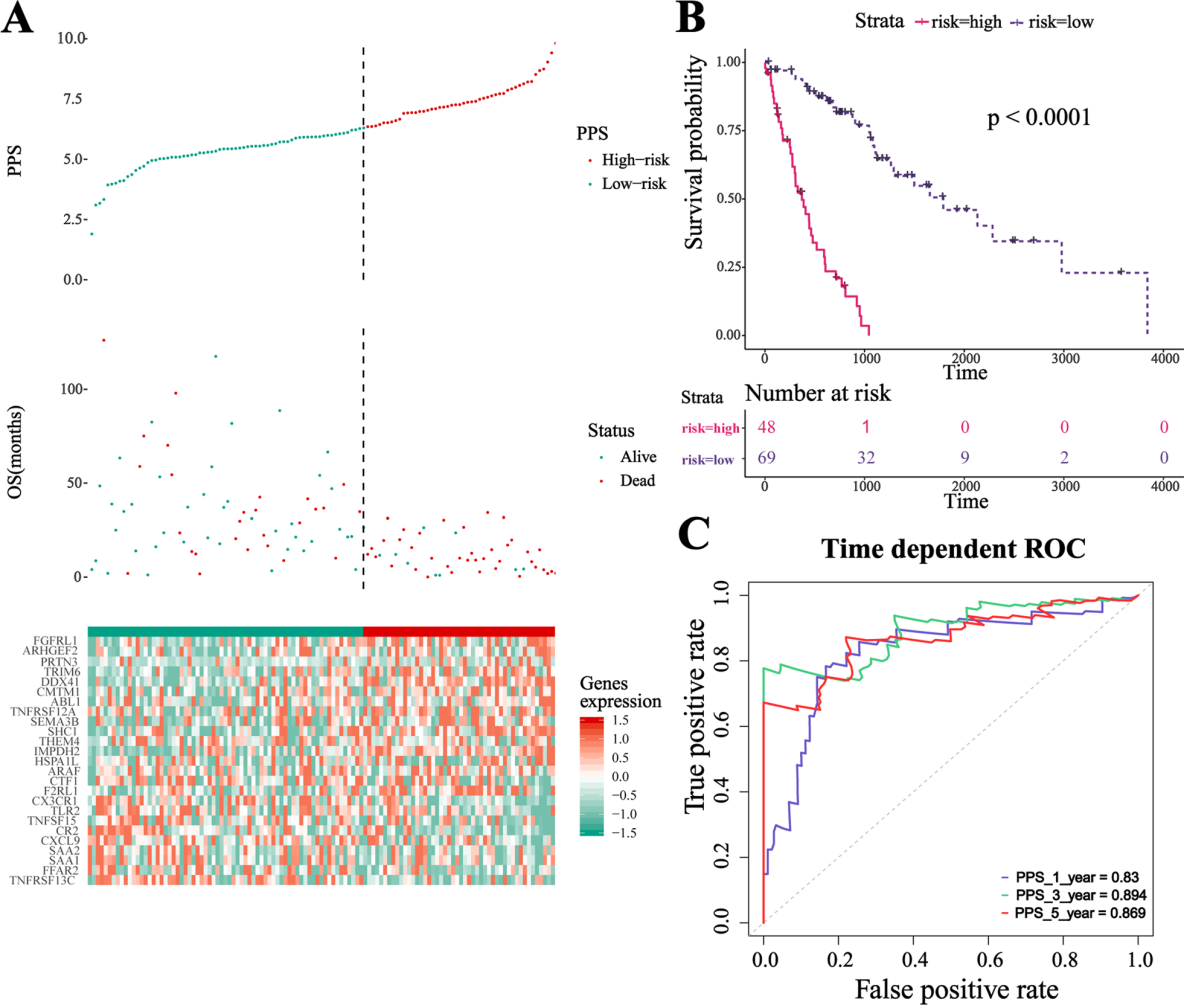
Construction of immune-related poor prognosis signature. **A** The distribution of PPS score, OS, and expression patterns of genes involved in the signature. **B** Kaplan–Meier curve of OS among PPS-high and PPS-low group patients. Log-rank statistic was conducted to test statistical significance. **C** Performance assessment of the PPS by AUC. ROC analysis revealed the AUC was 0.83 at 12 months, 0.894 at 36 months, and 0.869 at 60 months

We sought to establish a poor prognostic signature by using the samples' immune status. The samples in TCGA cohorts were randomly separated into training cohort (n = 117) and validation cohort (n = 78). We collected immune-related genes (from InnateDB and Immport databases), immune-related signatures (from MsigDB and previous studies), immune-related therapeutic signature (from Jiao Hu et al. study), immune-related scores (calculated by ESTIMATE algorithm), and abundance of TIICs (calculated by CIBERSORT algorithm). Feature engineering was conducted to filter OS-unrelated and cluster2-irrelevant variables (Additional file 3: Table S7-9. Firstly, the univariate cox test was conducted to seek out features that were associated with overall survival outcome. Then, the Wilcoxon test was used to find out features related to cluster-2. The features obtained finally were used in the PPS model construction). Then 25 gene-based LASSO-COX model was constructed, which we defined as PPS (Additional file 3: Table S10). PPS for each patient was calculated and patients were classified into high/low-risk groups according to the optimal cut-off determined by X-tile software (Fig. 3A). It could be observed that patients in the PPS-low group had a distinct survival advantage (log-rank test, p < 0.001, Fig. 3B) as compared to the PPS-high group. AUC of the PPS prediction for overall survival was 0.830 at 12 months, 0.894 at 36 months, and 0.869 at 60 months in the training cohort (Fig. 3C), which showed quite a good prediction efficiency. The same results were shared in the validation cohort (Additional file 1: Figure S4). Kaplan–Meier curves showed patients in PPS-low had a better overall survival (log-rank test, p = 0.004, Additional file 1: Figure S4A), and AUC of PPS prediction for OS was 0.725 at 12 months, 0.681 at 36 months, and 0.621 at 60 months (Additional file 1: Figure S4B). In addition, PPS was confirmed to be an independent prognostic factor both in the training and validation cohorts (Table 2). Then, we validated the PPS with two external data sets (Additional file 1: Figure S4), and the results were consistent with expectations (Additional file 1: Figure S4C-H).

**Table 2:** Univariate and multivariate analyses of clinicopathological characteristics and PPS with overall survival in training and validation cohort

	Univariate analysis			Multivariate analysis		
	HR	95% CI	*P* value	HR	95% CI	*P* value
*Training (n* = *117)*						
Age	1.003	0.976–1.032	0.812	1.029	0.998–1.060	0.067
pathologic_N	0.936	0.683–1.282	0.680	1.292	0.909–1.836	0.153
pathologic_T	1.269	0.967–1.664	0.086	1.228	0.893–1.690	0.207
Gender	1.600	0.945–2.709	0.080	0.983	0.550–1.757	0.954
PPS*	2.714	2.108–3.495	** < 0.001**	2.902	2.202–3.825	** < 0.001**
*Test (n* = *78)*						
Age	0.998	0.965–1.032	0.886	1.008	0.972–1.044	0.679
pathologic_N	1.207	0.822–1.771	0.337	1.346	0.823–2.199	0.236
pathologic_T	1.042	0.761–1.427	0.797	1.224	0.848–1.766	0.280
Gender	0.692	0.370–1.296	0.250	0.583	0.306–1.113	0.102
PPS*	1.694	1.272–2.256	** < 0.001**	1.718	1.289–2.288	** < 0.001**

The significant *P* value was indicated in bold

* Statistically significant results (*P*<0.05)

According to previous studies, several prognostic models have been proposed based on NSCLC, lung adenocarcinoma, or lung squamous cell carcinoma [45–50]. However, there was almost no signature proposed based on advanced NSCLC, and the actual use of the former models might lead to fallacies due to this. In our study, the AUC of PPS was 0.784 at 12 months and 0.808 at 36 months, and 0.764 at 60 months in our entire cohort (N = 195) (Additional file 1: Figure S4I-J), and the AUC of PPS in the luad/lusc subgroup were shown in the figure (Additional file 1: Figure S4K-L, LUAD: 0.802 at 12 M, 0.806 at 36 M, 0.727 at 60 M; LUSC: 0.757 at 12 M, 0.812 at 36 M, 0.788 at 60 M). Here, we compared the efficiency of our PPS model with other models and evaluated the AUC as a measure of accuracy. As shown in the table (Table 3, Additional file 1: Figure S4M-R), the PPS model always reached the highest AUC whether in advanced NSCLC, adenocarcinoma, or squamous, suggesting that our PPS had favorable efficacy for predicting overall survival in advanced NSCLC.

**Table 3:** Comparison of the performance of PPS with other previous signatures

	NSCLC	Adenocarcinoma				Squamous				Pubmed ID	Study subjects
	1 year	3 year	5 year	1 year	3 year	5 year	1 year	3 year	5 year		
PPS	0.784	0.808	0.764	0.802	0.806	0.727	0.757	0.812	0.788	NULL	Stage 3 & 4 NSCLC
Jia Li, et al	0.596	0.539	0.415		NULL			NULL		32,020,214	NSCLC
Jie Yao, et al	0.629	0.633	0.564		NULL			NULL		33,403,045	NSCLC
Han Wang, et al		NULL		0.614	0.489	0.445		NULL		32,989,393	LUAD
Jie Zhu, et al				0.587	0.594	0.691		NULL		32,695,805	LUAD
Deng gang Fu, et al		NULL			NULL		0.596	0.694	0.631	33,005,178	LUSC
Jili Hou, et al	NULL				NULL		0.593	0.537	0.593	33,466,167	LUSC

### The PPS score predicts immunotherapeutic benefits

To explore the biological significance of the PPS, the correlations between PPS and immune-related parameters were analyzed. Among 8 main TIICs, PPS was found to be positively correlated with M0 and M2 macrophages, and negatively correlated with CD8 + T cells, Tfh, activated CD + T memory cells, Tgd, M1 macrophage, and plasma (Additional file 1: Figure S5). In addition, PPS was negatively correlated with the majority of immunomodulatory factors. Notably, PPS was positively correlated with the expression of TGFβ/EMT-pathway-related molecules (COL4A1, ZEB1, ACTA2, TWIST1, VIM, TGFBR2), and several immunotherapy-associated signatures (Additional file 1: Figure S6). GSEA results (Additional file 1: Figure S7) revealed that many immune-related functions or pathways were enriched in the PPS-low group (such as “Adaptive_immune_response”, “Inflammatory_response”, “T_cell_receptor_signaling_pathway” and “B_cell_receptor_signaling_pathway”).

**Fig. 4:**
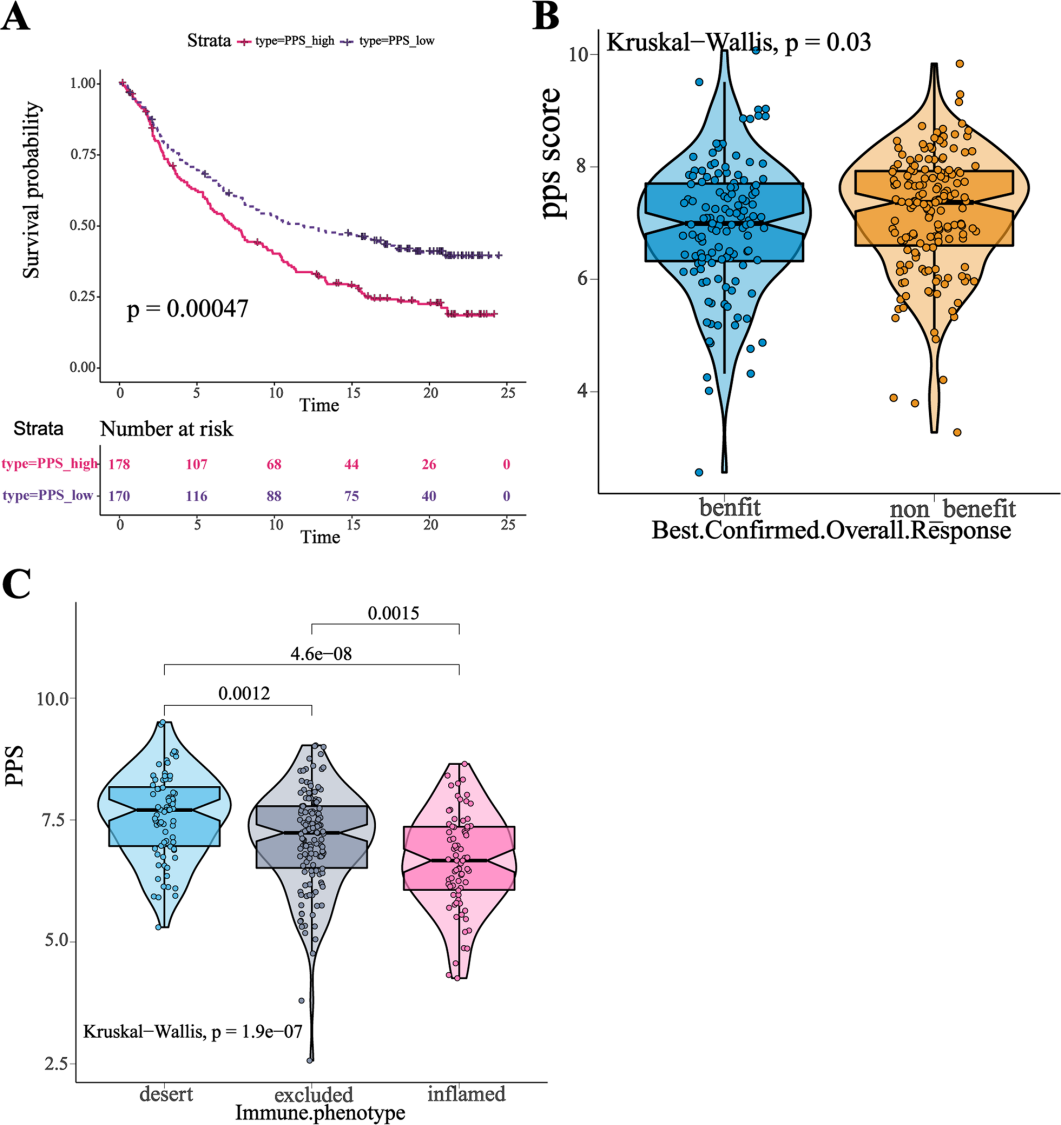
The performance and distribution of PPS in IMvigor210 cohort. **A** Kaplan–Meier curve of patients in PPS-high and PPS-low groups. Log-rank test statistic was conducted to test statistical significance. **B** The PPS distribution of patients in treatment-benefit and treatment-non-benefit groups. Kruskal–Wallis statistic was conducted to test statistical significance. **C** The PPS distribution of patients in different immune phenotype groups. Kruskal–Wallis statistic was conducted to test statistical significance

In the subsequent analysis, we evaluated the prognostic value of the PPS in three independent ICI immunotherapy cohorts (GSE126044 n = 16, GSE135222 n = 27, and IMvigor210 n = 348). Patients were assigned to PPS-high or PPS-low group. The survival outcome and distribution of the PPS in GSE126044 and GSE135222 cohorts were shown in the supplementary figures (Additional file 1: Figure S7D-G). However, the results were not statistically significant, which might be due to the small sample sizes. The patients who received anti-PD-L1 treatment in IMvigor210 were assigned to PPS-low or PPS-high groups, too. It was shown that the PPS-low group had a distinct overall survival advantage (log-rank test, *p* < 0.001, Fig. 4A). Patients benefited from the treatment (CR/PR/SD) tended to have lower PPS score as compared to those PD patients (Fig. 4B). Notably, the PPS score gradually decreased from immune-desert phenotype to immune-excluded phenotype to immune-inflamed phenotype (Fig. 4C). Overall, PPS score might have the prediction ability in patients treated with anti-PD(L)1, and a higher PPS score always associated with worse clinical outcome. The results were not statistically significant (*P* > 0.05) in GSE126044 and GSE135222 cohorts, which were likely to be ascribed to the small sample sizes.

### A robust model predicts sub-clusters based on immunological parameters

To build a classifier that could distinguish different subtypes for advanced NSCLC, we applied four algorithms (RF, SVM, Xgboost, Adaboost) to build the model, and selected the best one. Candidate variables included immune-related signatures, immune-related therapeutic signature, immune-related scores (calculated by ESTIMATE and IPS), PPS scores, and abundance of TIICs, and different clusters were set as response variables. Accuracy, precision, recall, F1 score, and AUC value in the validation cohort were used to measure the efficacy of different classifiers. Before the calculation, we adjusted the parameters used in different algorithms according to grid search or other approaches (Additional file 1: Figure S8A-B). Classifiers’ performance was shown in the table (Additional file 3: Table S11, Additional file 1: Figure S8C). The results indicated that the classifiers built by RF and Adaboost had higher efficacy than others, and Adaboost seems to be better. For example, the accuracy for cluster 1–3 was0.923, 0.936, 0.987 and AUC for cluster 1–3 was0.928, 0.896, 0.992 in RF, while in SVM, the accuracy for cluster 1–3 was 0.859, 0.872, 0.885, and AUC for cluster 1–3 was 0.864, 0.826, 0.835 (Additional file 3: Table S11). The detailed information of these classifiers was uploaded into Github (https://github.com/LClungcancer/nsclc-2021_classifier). The ranking plot of variables weight indicated that CD8 + T cell and Macrophages might be the keys to distinguish different clusters in patients with advanced NSCLC (Additional file 1: Figure S8D-E).

To verify the generalization ability of our classifiers, we test the performance of the selected two classifiers (RF and Adaboost) in the combined-affy cohort and combined-illumina cohort. The same KM clustering in the testing cohort was conducted, and we used Submap (GenePattern “Submap” module) to prove the identity of the clusters was the same as the TCGA cohort. Then, we test the performance of the selected two classifiers we constructed before. The result was shown in Additional file 3: Table S12, the classifiers showed good generalization ability (Additional file 3: Table S12). In addition, we used a neural network (NNet) to learn this classification. As shown in the Additional file 3: Table S13, “T cell CD8”, “T cell CD4 memory resting”, “Macrophage M0” and “B cell plasma” was the important variables in the classification, which was similar to the results of machine learning. The validation and Nnet procedure were uploaded to https://github.com/LClungcancer/nsclc-2021_classifier.

### Differences in somatic mutations related to the different clusters

**Fig. 5:**
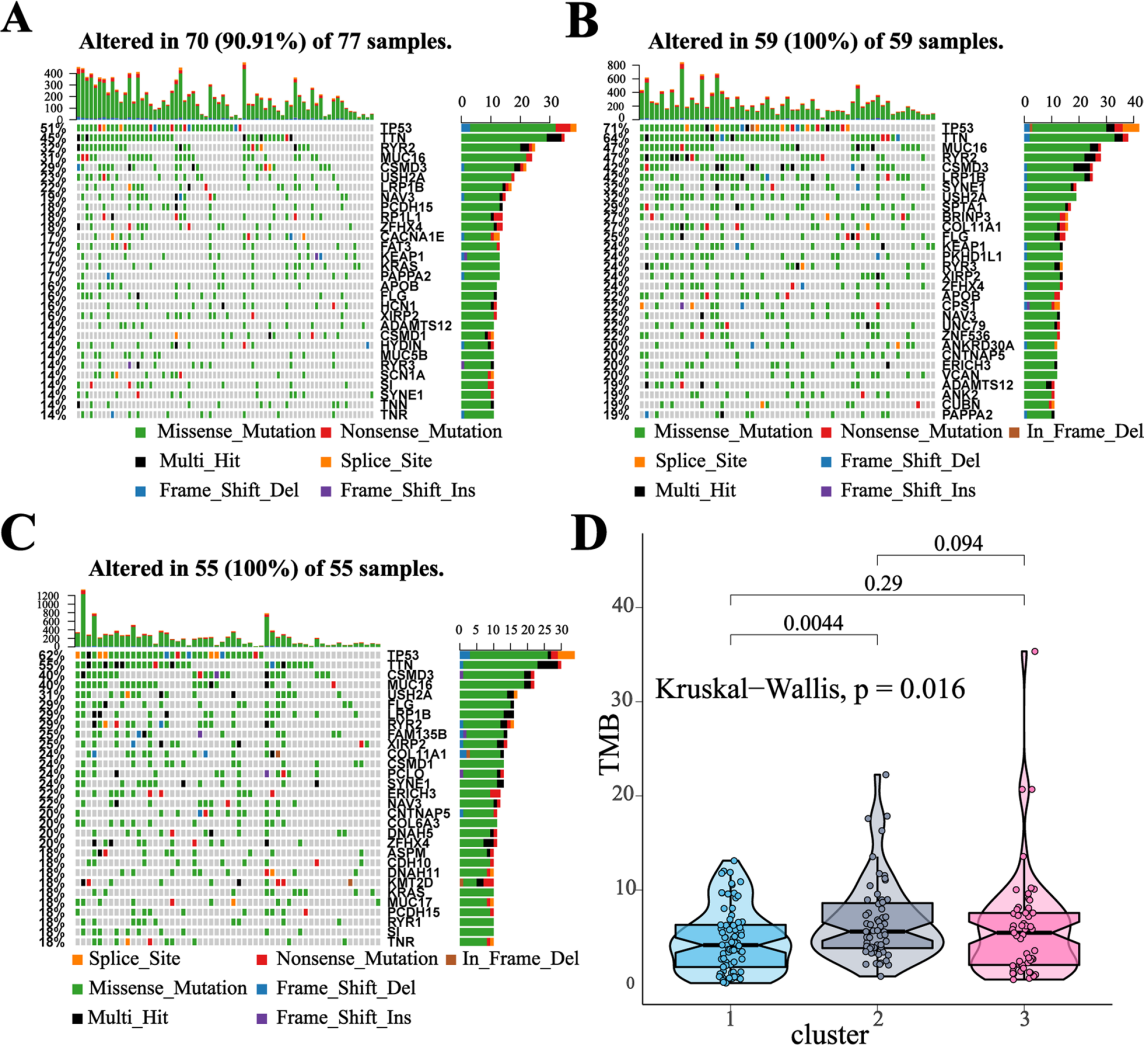
The landscape of mutation status among different clusters. **A**–**C** Top 30 genes with the highest mutation frequencies in cluster-1 (**A**), cluster-2 (**B**) and cluster-3 (**C**). **D** Tumor mutation burden (TMB) distribution in different clusters. Kruskal–Wallis statistic was conducted to test statistical significance

To reveal the relevant genetic alterations, we analyzed the somatic mutations among different clusters (Fig. 5A–C). Total tumor mutation burden (TMB) was higher in cluster 2 as compared to cluster 1 (Fig. 5D), while TMB showed no difference between cluster 2 and cluster 3 (Kruskal–Wallis test, *p* = 0.094). We further analyzed the mutation situations of the top 30 genes with the highest mutant frequency (Additional file 3: Table S14-15), and selected several high-frequency mutated genes in each cluster (including LRP1B, CSMD3, RYR2, RYR3, SYNE1, TTN). In addition, we collected some cancer drive genes and immunotherapy-related genes (including EGFR, ALK, KRAS, TP53, MUC16, MET, BRCA1, BRCA2, POLE, POLD1, MSH2, STK11, BRAF, PIK3CA, HER2, FGFR1, ROS1) [51]. Combined with high-frequency mutated genes we identified before, we examined the mutation proportion of these 23 genes among different clusters. The Chi-square test result revealed that TP53, MUC16, LRP1B, SYNE1, and TTN showed higher mutation proportion in cluster 2 as compared to cluster 1, and EGFR, RYR2 showed a higher proportion in cluster 2 as compare to cluster 3 (Additional file 3: Table S16).

### Genetic and epigenetic regulation related to the different clusters

To obtain a profound understanding of the difference among different clusters, we assessed somatic copy number alterations, DNA methylation, and miRNA for these three clusters. Precisely, we made two comparisons (C2 vs C1, C2 vs C3). First, differentially expression genes (DEGs) between cluster-2 and cluster-1 or between cluster-2 and cluster-3 were analyzed. In the comparison between C2 and C1, 2318 DEGs were identified, including 2135 genes with a higher expression in cluster-1 and 183 genes with a higher expression in cluster-2. In the comparison between C2 and C3, 1242 DEGs were identified, including 1001 genes with a higher expression in cluster-1 and 241 genes with a higher expression in cluster-2 (Additional file 3: Table S17).

**Fig. 6:**
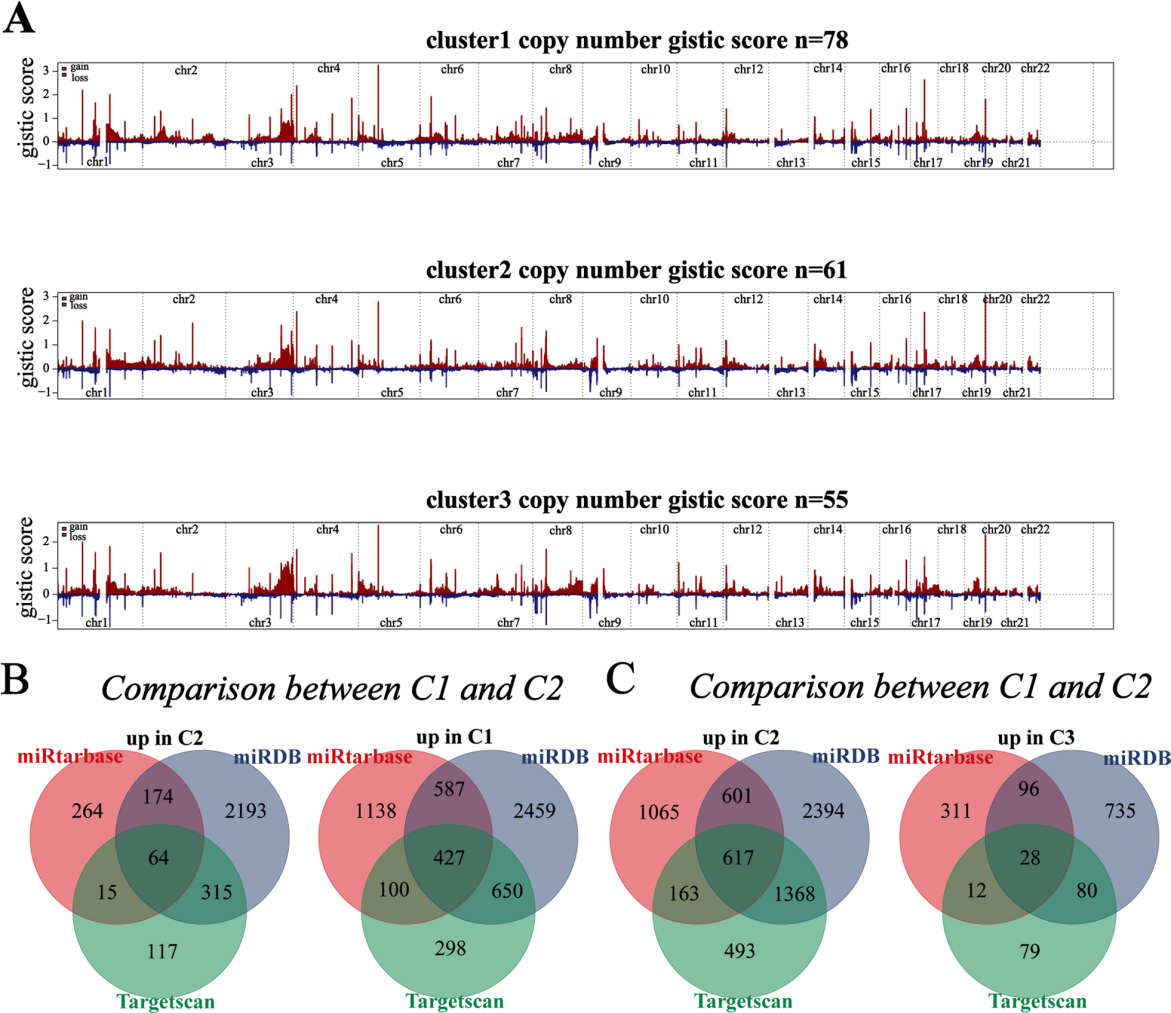
The differences in epigenetic regulation in different clusters. **A** Comparison of the copy number alterations among different clusters. Gistic scores were assessed by GISTIC 2.0 with red for amplification and blue for deletion. **B** In the comparison between cluster-1 and cluster-2, venn diagram summarizes the DEmiRNA-mRNA links predicted by miRtarbase, miRDB and Targetscan databases. **C** In the comparison between cluster-3 and cluster-2, venn diagram summarizes the DEmiRNA-mRNA links predicted by miRtarbase, miRDB and Targetscan databases

SCNAs are widespread in human cancers and have a profound impact on immune evasion. GISTIC 2.0 was used to conduct genomic variation analysis (Fig. 6A). In the comparison between C2 and C1, 523 DEGs upregulated in cluster-1 were encoded by the genomic region with a higher frequency for deletions in cluster-2 or copy number gains in cluster-1; 71 DEGs upregulated in cluster-2 were encoded by the genomic region with a higher frequency for deletions in cluster-1 or copy number gains in cluster-2 (Additional file 3: Table S18). In the comparison between C2 and C3, 230 DEGs upregulated in cluster-3 were encoded by the genomic region with a higher frequency of deletions in cluster-2 or copy number gains in cluster-3; 17 DEGs upregulated in cluster-2 were encoded by the genomic region with a higher frequency of deletions in cluster-3 or copy number gains in cluster-2 (Additional file 3: Table S18).

To assess the impact of DNA methylation among different clusters, DNA methylation data (Illumina Human Methylation 450 k) were analyzed. In the comparison between C2 and C1, 7 probes with higher beta values in cluster-1 were located in the proximal promoter of DEGs upregulated in cluster-2, while 10 probes with higher beta values in cluster-2 were located in the proximal promoter of DEGs upregulated in cluster-1. In the comparison between C2 and C3, 1 probe with higher beta values in cluster-3 were located in the proximal promoter of DEGs upregulated in cluster-2, while 17 probes with higher beta values in cluster-2 were located in the proximal promoter of DEGs upregulated in cluster-3 (Additional file 3: Table S19).

Next, we identified differentially expressed miRNA between C2 and C1 or between C2 and C3. In the comparison between C2 and C1, 16 miRNA were upregulated in C1 and 52 miRNA were upregulated in C2; In the comparison between C2 and C3, 54 miRNA were upregulated in C3 and 9 miRNA were upregulated in C2. We examined the reliable links between DEmiRNAs and DEGs based on three databases (miRDB, miRtarbase, Targetscan, prediction in at least two databases was considered reliable). In the comparison between C2 and C1, DEmiRNAs upregulated in cluster-1 target 3 DEGs in cluster-2, and DEmiRNAs upregulated in cluster-2 target 314 DEGs in cluster-1 (Fig. 6B, Additional file 3: Table S20). In the comparison between C2 and C3, DEmiRNAs upregulated in cluster-3 target 7 DEGs in cluster-2, and DEmiRNAs upregulated in cluster-2 target 14 DEGs in cluster-3 (Fig. 6C, Additional file 3: Table S20).

### Key DEGs affected by genetic and epigenetic regulation

We assumed that DEGs affected by different genetic and epigenetic regulation might play an important role in the transformation of the phenotype. Genes identified in at least two out of three above analyses (SCNA, DNA methylation, and miRNA) were considered key DEGs. In the comparison between C2 and C1, 84 key DEGs were identified (80 key DEGs upregulated in cluster-1 and 4 key DEGs upregulated in cluster-2, Additional file 1: Figure S9A). The PPI network was constructed based on the 84 key DEGs using the STRING database, and the result highlighted HSPA8, CREB1, RAP1A as the key nodes within the network (Additional file 1: Figure S9C). In the comparison between C2 and C3, 5 key DEGs were identified (including GRM2, TBXA2R, PLEC, LUZP1, RELA, all 5 key DEGs were upregulated in cluster-3, Additional file 1: Figure S9B). These results indicated that HSPA8, CREB1, RAP1A might be the potential therapeutic targets for patients in cluster-1. GRM2, TBXA2R, PLEC, LUZP1, RELA might be associated with poor prognosis in cluster-3.

### Identification of potential drugs for patients in different clusters

After preprocessing, drug sensitivity profiles of 1291 compounds in PRISM dataset and 354 compounds in CTRP were used for subsequent analysis. The drug sensitivity of entire clinical samples was predicted based on a ridge regression model (“pRRophetic” package in R), and we obtained the AUC value of each compound in each sample (lower AUC values indicate increased sensitivity to specific compounds). We assessed the compounds with higher sensitivity in cluster-1, cluster-2, cluster-3 in turn, and these analyses were conducted using CTRP and PRISM data, respectively.

**Fig. 7:**
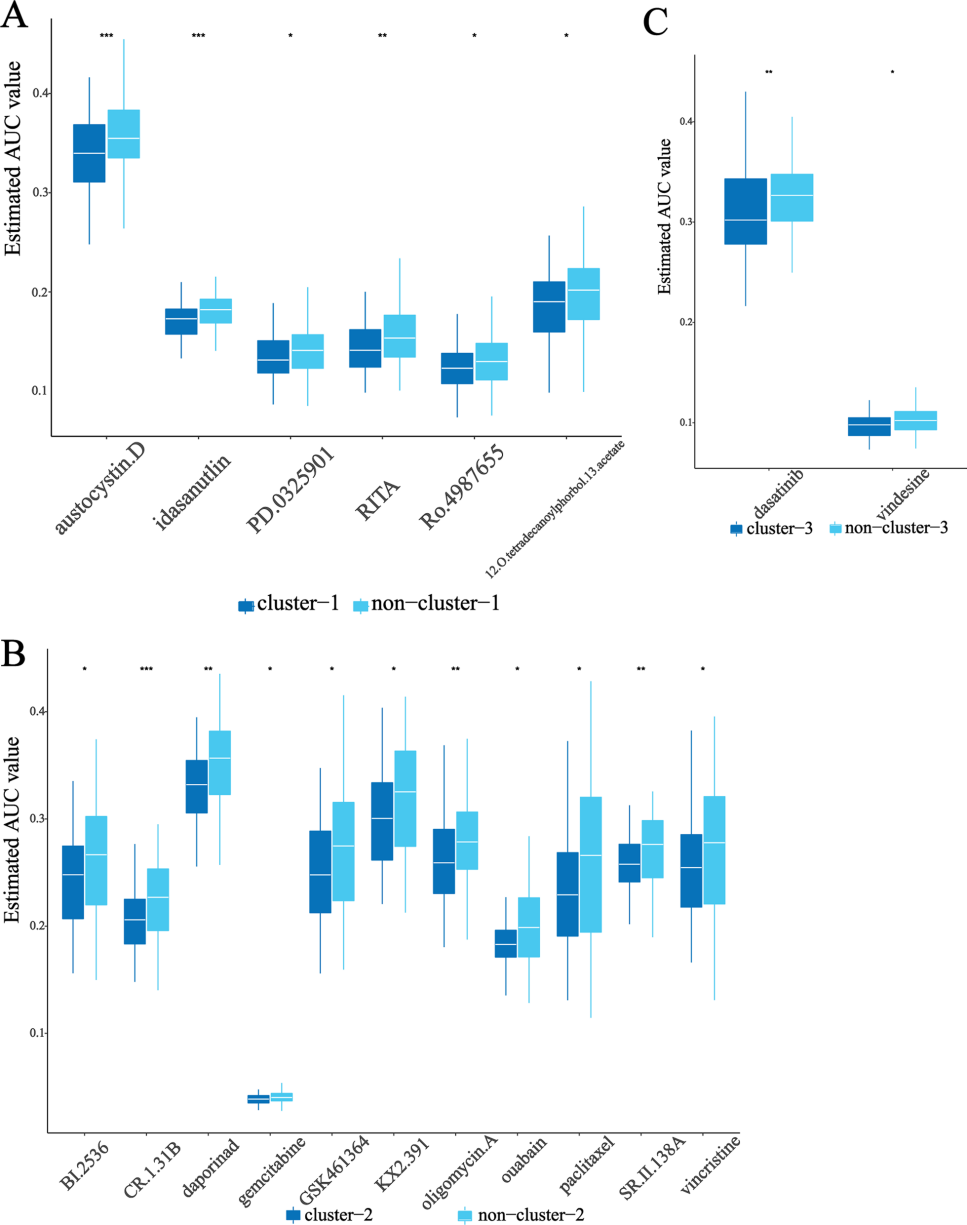
Identification of potential agents in each cluster. Differential drug response analysis of compounds identified in cluster-1 (**A**), cluster-2 (**B**), and cluster-3 (**C**). Note that higher estimated AUC values imply lower drug sensitivity. ***, *P* < 0.001; **, *P* < 0.01; *, *P* < 0.05

Compounds with lower AUC values in specific clusters were identified (Log2FC > 0.07, *p* value < 0.05, Fig. 7, Additional file 3: Table S21). For cluster-1 (Fig. 7A), 5 PRISM-derived compounds (including RITA, 12-O-tetradecanoylphorbol-13-acetate, Ro-4987655, idasanutlin, PD-0325901) and 1 CTRP-derived compounds (including austocystin D) were identified; For cluster-2 (Fig. 7B), 1 PRISM-derived compounds (including gemcitabine) and 10 CTRP-derived compounds (including paclitaxel, CR-1-31B, GSK461364, BI-2536, vincristine, oligomycin A, ouabain, KX2-391, SR-II-138A, daporinad) were identified; For cluster-3 (Fig. 7C), 1 PRISM-derived compounds (including vindesine) and 1 CTRP-derived compounds (including dasatinib) were identified. All compounds identified had lower AUC values in a specific cluster as compared to the other clusters. These compounds might hold therapeutic potential in patients with advanced NSCLC of different clusters.

## Discussion

Despite substantial advances having been made in the treatment of lung cancer within the past few decades, the therapeutic outcome of advanced NSCLC remains far from satisfactory [52]. In this study, various machine learning algorithms and bioinformatic analysis were conducted to depict landscapes of patients with advanced NSCLC. The landscape of cancer research and treatment is gradually changing with the pervading of AI (Artificial Intelligence). The frontier of cancer research involves collaborations between medical oncologists and computer scientists. Specifically, with the application of ML (machine learning), DL (deep learning), and multiple neural networks, many issues have been addressed, especially the diagnosis and prognosis prediction of cancer [53, 54]. In recent years, AI has provided a new approach for the diagnosis and prognosis of cancer and made cancer prediction performance reach a new height [54]. According to Ahmed et al. [53], the use of AI on oral oncology is in the nascent stage, and research such as digital histopathologic images is very few, indicating that we should focus on cancer at more levels. In our study, we focused on the NSCLC patients at an advanced stage. When applying ML, we used multiple methods (e.g. four MLs and a neural network were applied in the construction of the classifiers) and data from different sources (e.g. drug sensitivity data from CTRP and PRISM databases) to maximize the reliability. When we measured the performance of results, multiple indicators (e.g. accuracy, recall, precision, F1 score, and AUC were used to measure the performance of the classifier) and horizontal comparison (e.g. the PPS model was compared with the prediction model proposed by previous studies) were used to ensure the accuracy of the analysis. In general, our study was not just a “Training-Validation” pattern. We attempted to explore the issue from multi-method, multi-angle, and multi-measure.We acknowledged that the advanced NSCLC patients could be classified into three clusters, and each cluster has its characteristics: cluster-1 was characterized by increases in the infiltration of resting DCs, M2.marcrophages, activated mast cells, monocytes, activated NKs, and resting CD4 + T memory cells; cluster-2 was characterized by evident increase in the infiltration of plasma, M1.macrophages, activated CD4 + T memory cells, CD8 + T cells, T follicular helper cells, and Tregs; cluster-3 was characterized by high infiltration of M0.macrophages and resting mast cells and exhibited decreases in other TIICs. Different classifiers were then designed to distinguish different clusters based on various machine learning algorithms (including RF, SVM, Xgboost, and Adaboost), and RF/Adaboost were considered as highly efficient classifiers with the best performance. These analyses not only simplified the basis for the classification but ensured the accuracy of the classifier. CD8 + T cells and Macrophages were identified to play a major role in the classification. In other words, CD8 + T cells and Macrophages are the key TIICs to alter immune phenotypes in advanced NSCLC, which is in agreement with previous researches [55, 56]. In addition, cluster-2 was found to be correlated with better overall survival outcome and might have a better clinical response to immunotherapy.

We then constructed the Poor Prognosis Signature based on the immune-related parameters, and we found out that the PPS score had survival prediction efficacy in patients treated with anti-PD(L)1 immunotherapy. Similar, similar prediction models have been proposed in previous studies. But the actual use of them might lead to fallacies since almost none of them were constructed based on the advanced tumor stage. The benchmarking results showed that our poor prognosis signature had the best prediction performance. To find out the key molecules in the differences between cluster-2 and cluster-1 or between cluster-2 and cluster-3, we turned to explore the genetic or epigenetic alterations among different clusters. The results unraveled that three key nodes (including HSPA8, CREB1, RAP1A) showed noteworthy differences between cluster-1 and cluster-2. Similarly, we found five molecules (including GRM2, TBXA2R, PLEC, LUZP1, RELA) that might be associated with poor prognosis in cluster-3. In previous studies, HSPA8 and RAP1A have been demonstrated to be associated with cancer growth and proliferation in various human cancers [57, 58]. CREB1 was considered to promote invasion and migration in human cancers, including NSCLC [59, 60]. In our analysis, we came to the point that these three molecules might serve as potential therapeutic targets for patients in cluster-1.

Finally, based on drug sensitivity data derived from CTRP and PRISM, we identified several compounds which might serve as medication for different clusters of patients with advanced NSCLC. Specifically, six compounds for cluster-1 (RITA, 12-O-tetradecanoylphorbol-13-acetate, Ro-4987655, idasanutlin, PD-0325901, austocystin D), 11 compounds for cluster-2 (gemcitabine, paclitaxel, CR-1-31B, GSK461364, BI-2536, vincristine, oligomycin A, ouabain, KX2-391, SR-II-138A, daporinad), and 2 compounds for cluster-3 (vindesine, dasatinib). These results gave us some clues. For example, MAP2K1 inhibitors (including PD-0325901 and RO-4987655) showed their capacity of improving PFS and OS of patients with solid tumors as well as the major treatment-related toxicity [61, 62]. Our study further unraveled that PD-0325901 or RO-4987655 might be more applicable to cluster-1 patients with advanced NSCLC. Common antitumor drugs, including gemcitabine and paclitaxel [63–65], might apply to cluster-2, and dasatinib might be more applicable to patients in cluster-3.

However, there are still shortcomings and a lot of room for improvement in our study. A limitation of the study is the small sample size. In our study, the sample size of the main cohort (TCGA cohort, N = 195) was small. However, there are not much data of advanced NSCLC available in the public database. Thus, we could only verify the PPS model and the performance of the classifier using small sample data. On the other hand, the epigenetic-related analysis could only be conducted in the main cohort due to the lack of relevant data in the other cohorts, which might cause a certain degree of bias. In future studies, we will take account of these factors to enhance our study. In addition, we only selected the most prominent shift in mutation or drug sensitivity for further analysis, which could cause a certain bias.

## Conclusions

In conclusion, our study established new stratification of stage 3–stage 4 NSCLC, simplified the classifications, built an immune-related poor prognosis signature, analyzed the key therapeutic targets in cluster1/3, and explored the potential drug for patients in each cluster. With the promotion of the precision medicine concept, our study could provide more convenience for diagnosis and treatment for patients with advanced NSCLC. There are also some limitations to this study. The verification of the conclusions needs to be determined in related clinical trials in the future.

## Supplementary Information

**Additional file 1**. Supplementary Figures (S1-10).

**Additional file 2**. Supplementary Tables (S1-6).

**Additional file 3**. Supplementary Tables (S7-21).

## Data Availability

TCGA dataset was downloaded from Xena (https://xenabrowser.net/datapages/,TCGA-LUAD, TCGA-LUSC). Multi-omics data was downloaded from data portal (https://portal.gdc.cancer.gov/,TCGA-LUAD, TCGA-LUSC). Other transcriptome datasets including anti-PD(L)1 treatment cohorts were downloaded from Gene Expression Omnibus (http://www.ncbi.nlm.nih.gov/geo/,accession number: GSE37745, GSE29013, GSE42127, GSE41271, GSE135222, GSE126044) or Imvigor210 (http://research-pub.gene.com/IMvigor210CoreBiologies/. All data in this cohort was integrated into the R package “IMvigor210CoreBiologies”). Immune-related genes were obtained from InnateDB (https://www.innatedb.com) and Immport (https://www.immport.org/). Drug sensitivity data was obtained from PRISM (https://depmap.org/portal/prism/) and CTRP (https://portals.broadinstitute.org/ctrp). Our classifiers have been uploaded to Github, and can be found at (https://github.com/LClungcancer/nsclc-2021_classifier).

## Abbreviations

NSCLC: Non-small cell lung cancer
LUAD: Lung adenocarcinoma
LUSC: Lung squamous cell carcinoma
ICI: Immune checkpoint inhibitors
MSI: Microsatellite instability
CTLA-4: Cytotoxic T-lymphocyte antigen-4
PD-1: Programmed death 1
PD-L1: Programmed cell death ligand 1
LM22: 22 Immune cell types
SVM: Support vector machine
RF: Randomforest
Xgboost: EXtreme gradient boosting
Adaboost: Adaptive boosting
TMB: Tumor mutation burden
TIICs: Tumor-infiltrating immune cells
MHC: Major histocompatibility complex class
LASSO: Least absolute shrinkage and selection operator
CR: Complete response
PR: Partial response
SD: Stable disease
PD: Progressive disease
ROC: Receiver operating characteristic curve
AUC: The area under curve
