# Supplementary material for: Machine learning and bioinformatics analysis revealed classification and potential treatment strategy in stage 3–4 NSCLC patients
Source: BMC Med Genomics. 2022 Feb 22;15:33. doi: 10.1186/s12920-022-01184-1 (PMC8862473; doi:10.1186/s12920-022-01184-1)
Supplement: Supplementary file 1 — Additional file 1. Supplementary Figures (S1-10). [file 12920_2022_1184_MOESM1_ESM.pdf]

Figure S1

A

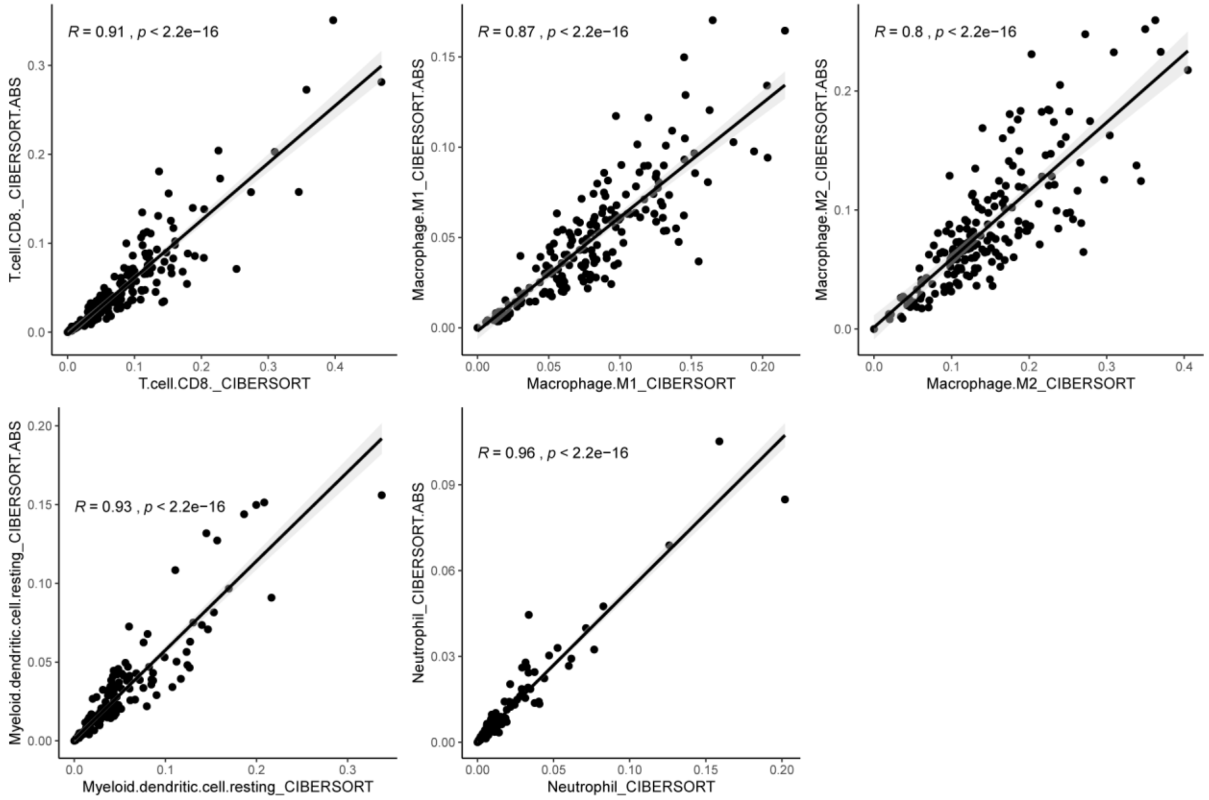

B

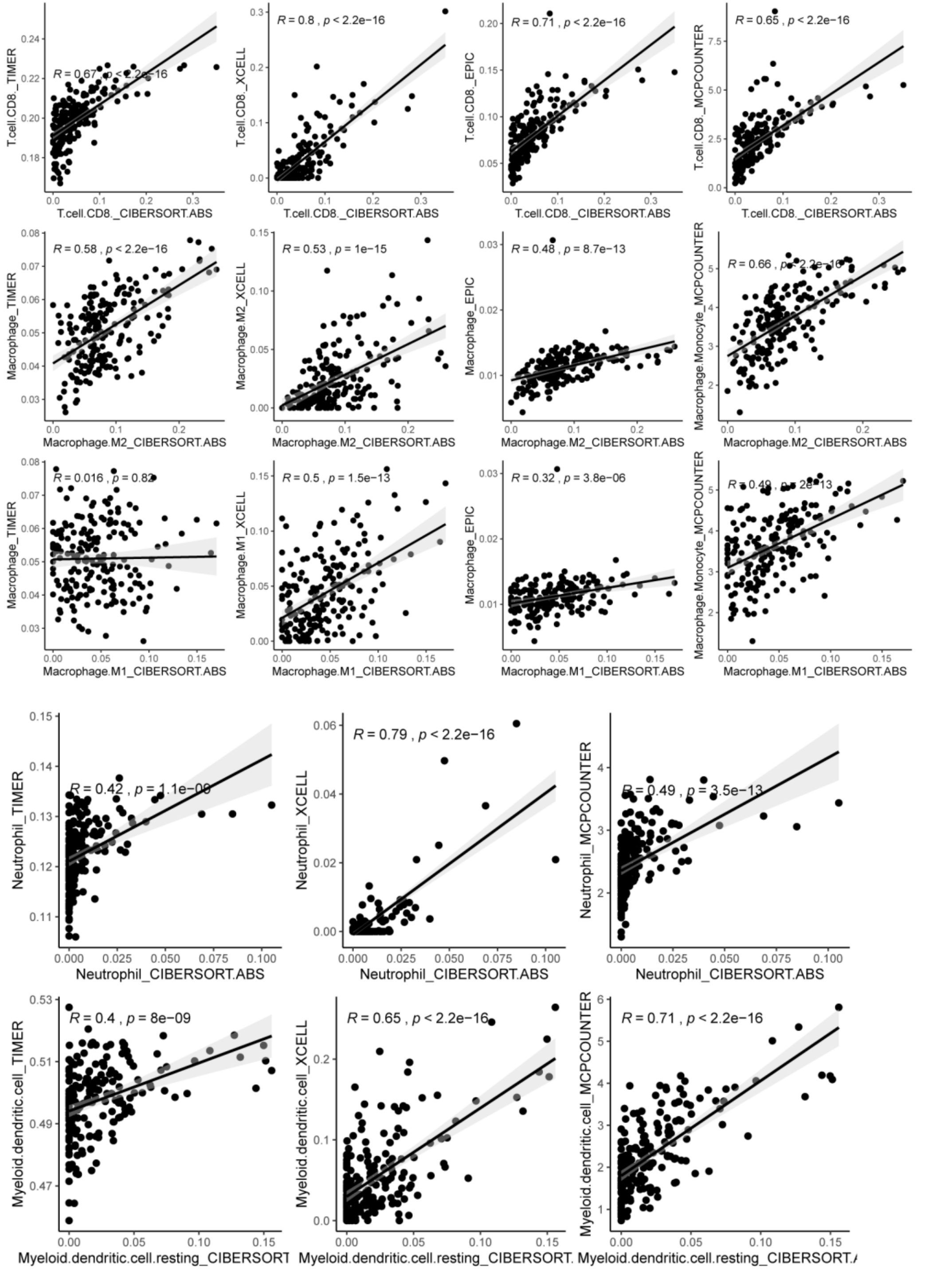

Fig S1. The correlation between CIBERSORT and other algorithms. (A). The correlation between the abundance of five TIICs (T cell CD8, M1.Macrophage, M2.Macrophage, Dendritic cell resting, Neutrophil) calculated by CIBERSORT relative mode and absolute mode. Correlation was analyzed by Spearman test. (B). The correlation between the abundance of common TIICs calculated by CIBERSORT (Absolute mode) and other algorithms (including TIMER, xCELL, EPIC, MCPcounter). Correlation was analyzed by Spearman test.

Figure S2

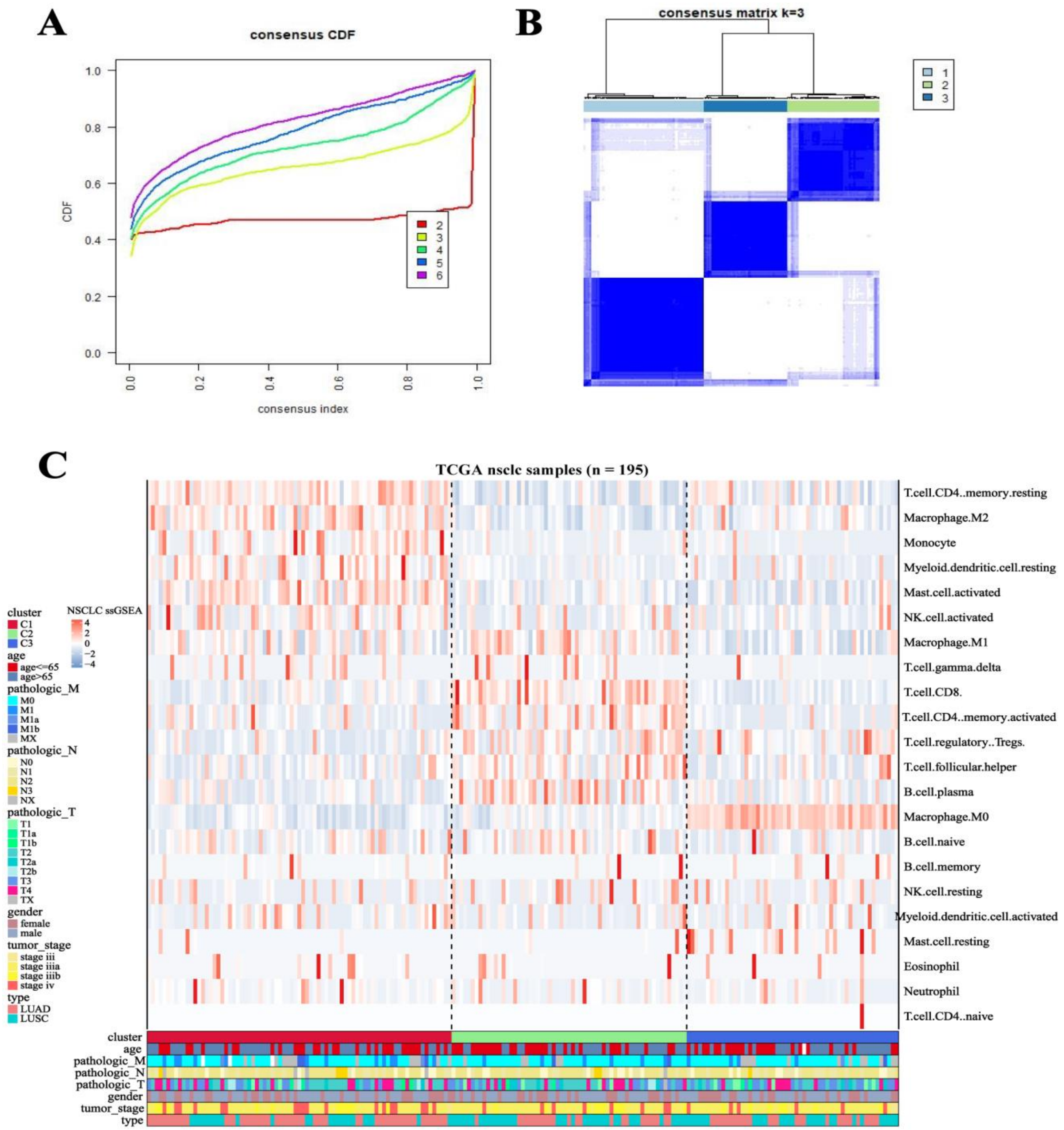

Fig S2. Unsupervised clustering of stage iii-iv NSCLC patients based on LM22. (A)-(B). Optimal number of cluster groups identified by unsupervised kmeans clustering. (C). K-means clustering based on LM22 abundance revealed three distinct clsuters. The heatmap was annotated by age, gender, NSCLC type, TNM stage and pathological stage.

Figure S3

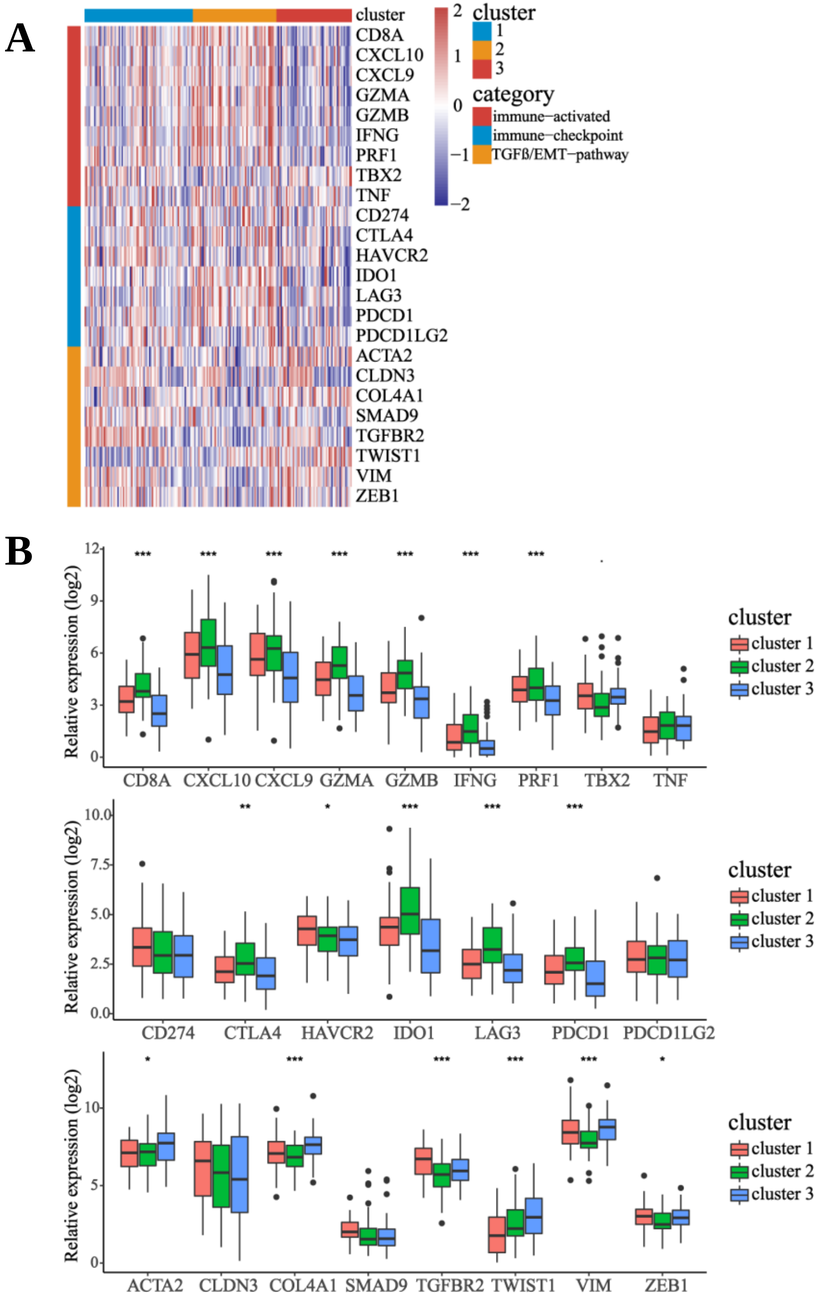

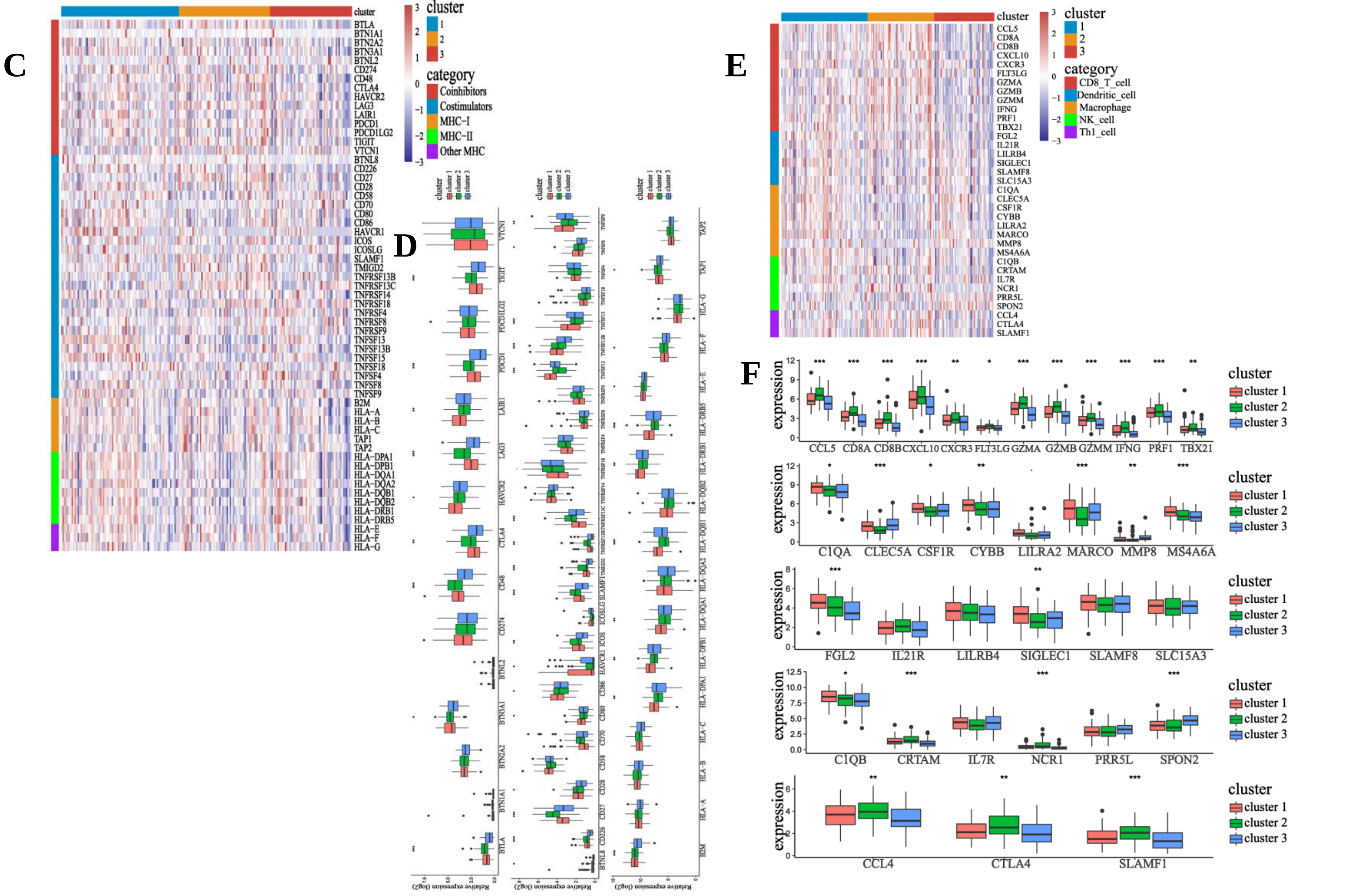

Fig S3. The expression of selected chemokine, cytokine, coinhibitory, costimulatory, MHC-related molecules, and effector genes of several TIICs in each cluster. (A). Heatmap revealed the distribution of immune-activated, immune checkpoint and TGFb/EMT-related molecules among different clusters. (B). Boxplot showed the difference of above molecules among different clusters in detail. ***, P < 0.0001; **, P < 0.001; *, 0.001 < P < 0.01. (C). Heatmap revealed the distribution of coinhibitory, costimulatory and MHC-related molecules among different clusters. (D). Boxplot showed the difference of above molecules among different clusters in detail. ***, P < 0.0001; **, P < 0.001; *, 0.001 < P < 0.01. (E). Heatmap revealed the distribution of effector genes of CD8+T cell, Dendritic cell, Macrophage, NK cell and Th1 cell among different clusters. (F). Boxplot showed the difference of above molecules among different clusters in detail. ***, P < 0.0001; **, P < 0.001; *, 0.001 < P < 0.01.

Figure S4

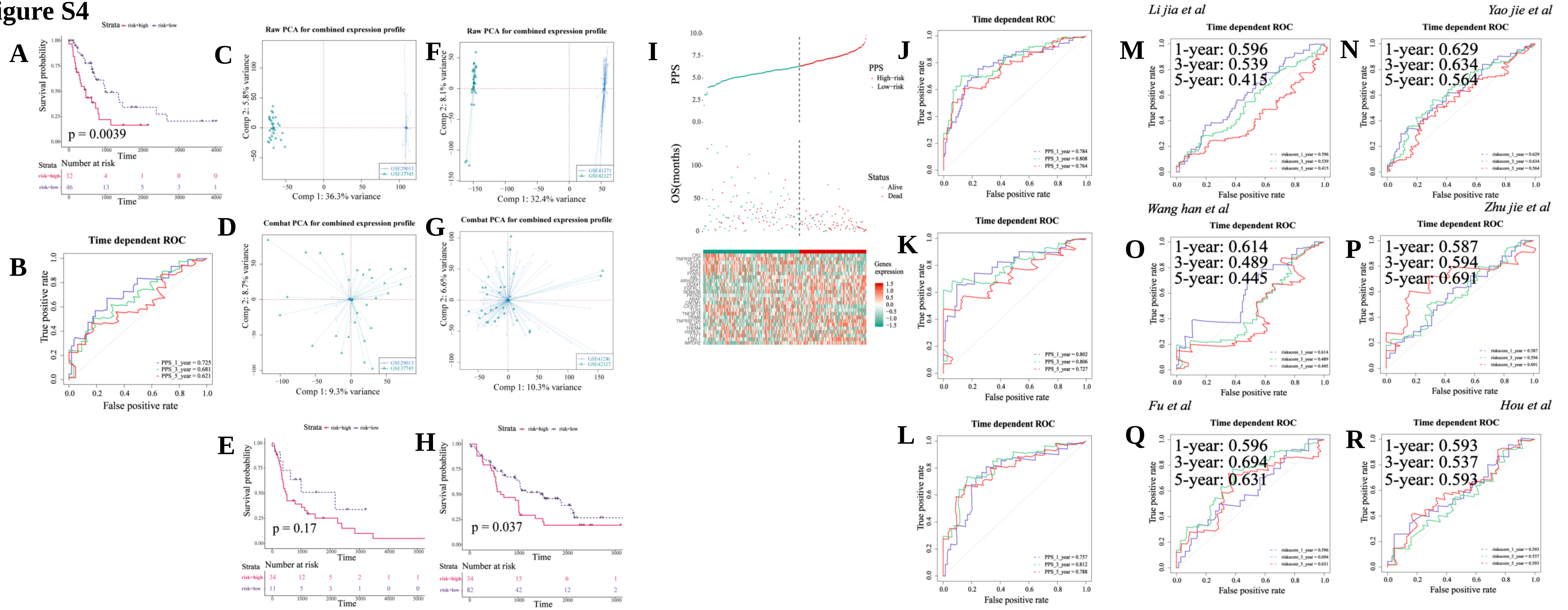

Fig S4. Validation of PPS score in validation and two external cohorts, and the performance of riskscore derived from previous studies in stage iii-iv TCGANSCLC cohort. (A) Kaplan-Meier curve of patients in PPS-low and PPS-high groups in TCGA-validation cohort. Log- rank statistic was conducted to test statistical significance. (B). Performance assessment of PPS by AUC in TCGA-validation cohort. ROC analysis revealed the AUC was 0.725 at 12 months, 0.681 at 36 months, and 0.621 at 60 months. (C) Batch effects examination between GSE29013 and GSE37745 before batch effect removal. (D). Batch effects examination between GSE29013 and GSE37745 after batch effect removal. (E). Kaplan-Meier curve of patients in PPS-low and PPS-high groups in affy cohort (GSE29013+GSE37745). Log- rank statistic was conducted to test statistical significance. (F). Batch effects examination between GSE41271 and GSE42127 before batch effect removal. (G). Batch effects examination between GSE41271 and GSE42127 after batch effect removal. (H). Kaplan-Meier curve of patients in PPS-low and PPS-high groups in illumina cohort(GSE41271+GSE42127). Log- rank statistic was conducted to test statistical significance. (I. The distribution of PPS score, OS, and expression of genes involved in the signature. (J). Performance assessment of PPS by AUC in entire cohort. ROC analysis revealed the AUC was 0.784 at 12 months, 0.808 at 36 months, and 0.764 at 60 months. (K). Performance assessment of PPS by AUC in entire LUAD cohort. ROC analysis revealed the AUC was 0.802 at 12 months, 0.806 at 36 months, and 0.727 at 60 months. (L). Performance assessment of PPS by AUC in entire LUSC cohort. ROC analysis revealed the AUC was 0.757 at 12 months, 0.812 at 36 months, and 0.788 at 60 months. (M)-(N): Performance assessment of riskscore (derived from Li jia et al (M) or Yao jie et al (N) studies) in entire stage iii-iv NSCLC cohort. (O)-(P): Performance assessment of riskscore (derived from Wang han et al (O) or Zhu jie et al (P) studies) in stage iii-iv LUAD cohort. (Q)-(R): Performance assessment of riskscore (derived from Fu et al (Q) or Hou et al (R) studies) in stage iii-iv LUSC cohort.

Figure S5

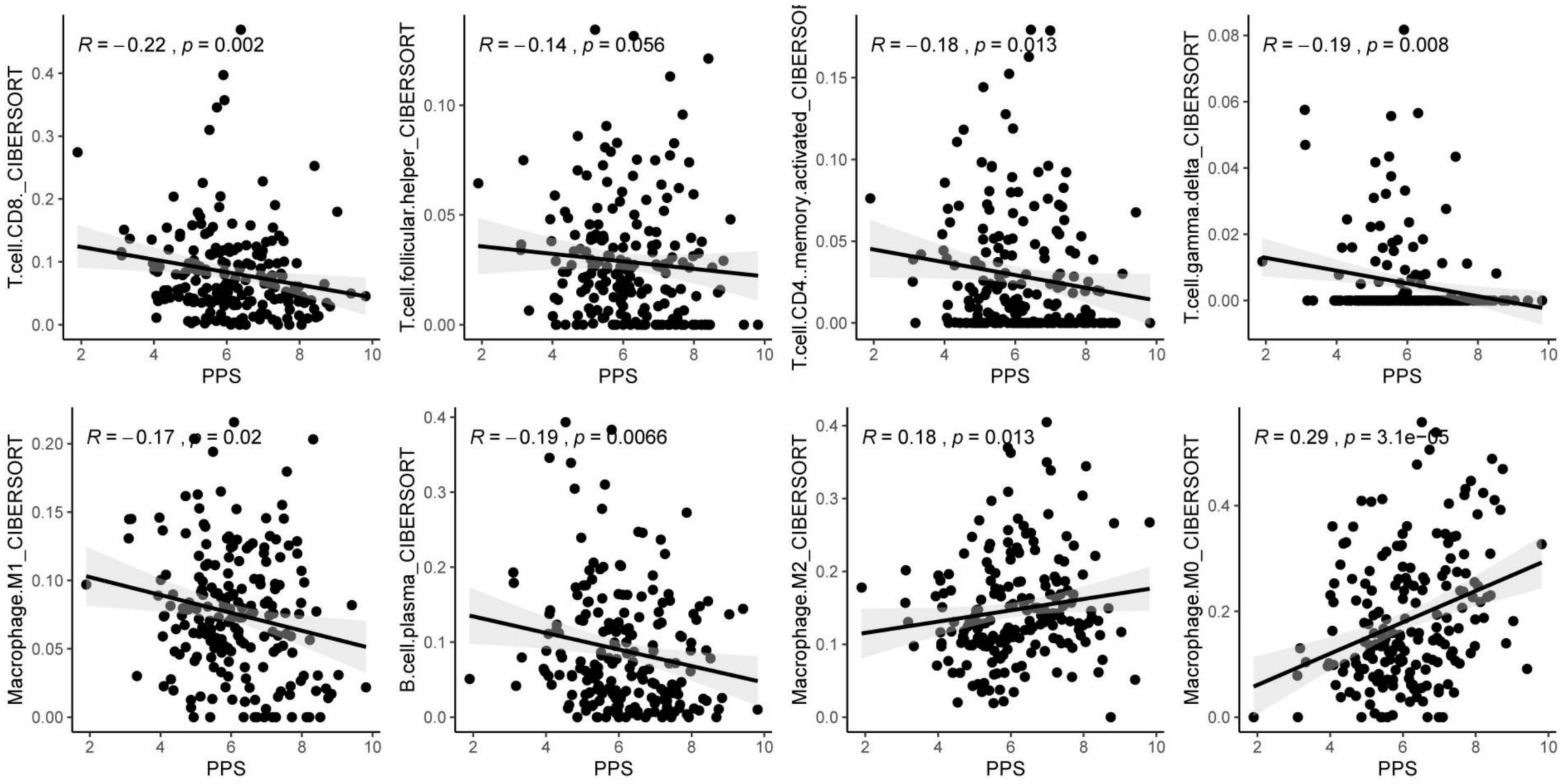

Fig. S5 The correlation between PPS score and the abundance of eight selected immune cells. The correlation between PPS score and eight TIICs (T cell CD8, T cell follicular helper, T cell CD4 memory activated, T cell gamma delta, M1.Macrophage, Plasma, M2.Macrophage, M0.Macrophage). Correlation was analyzed by Spearman test.

Figure S6

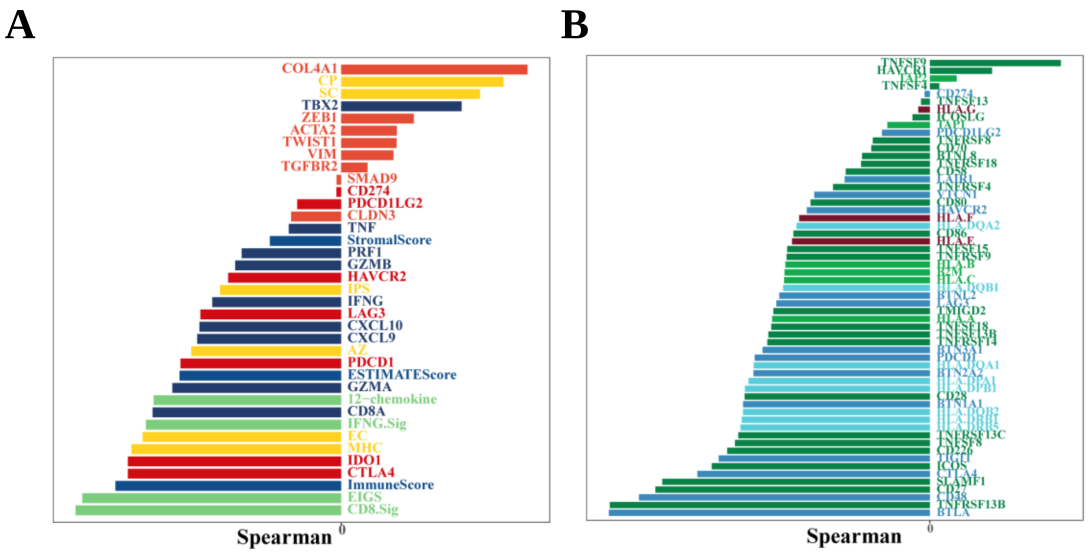

Fig S6. Correlation between PPS and immune-related molecules. Different colors represent different categories. Correlation was analyzed by Spearman test.

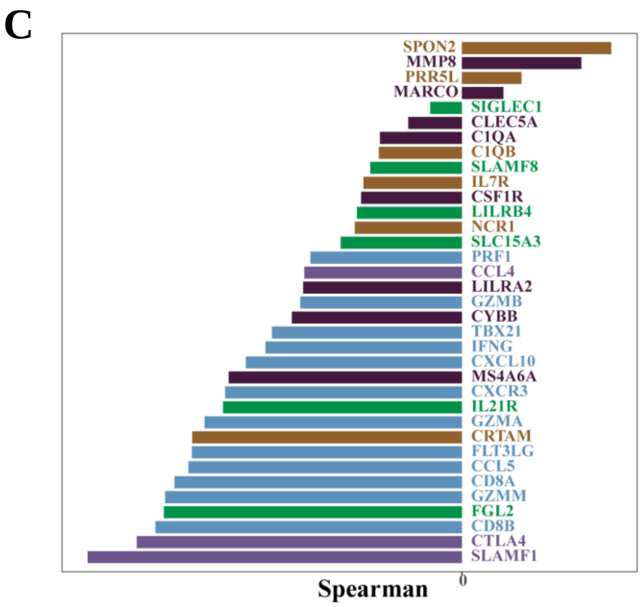

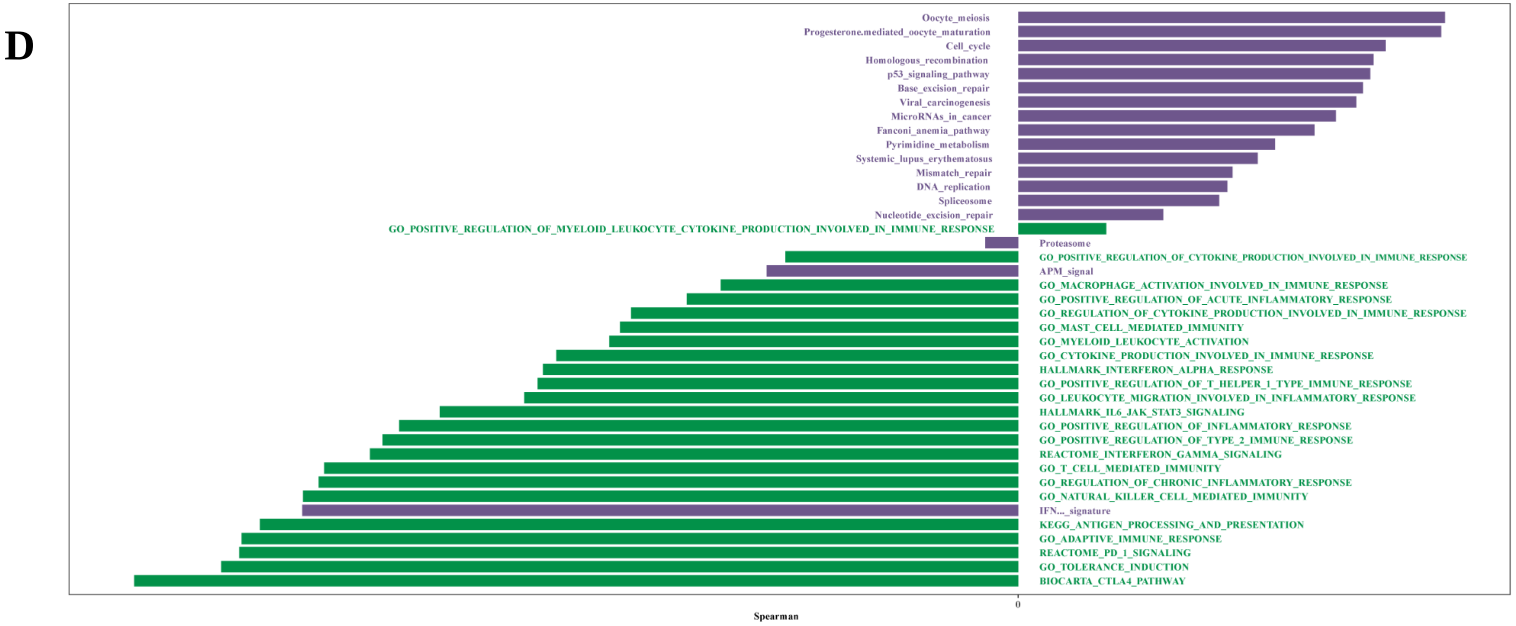

Figure S7

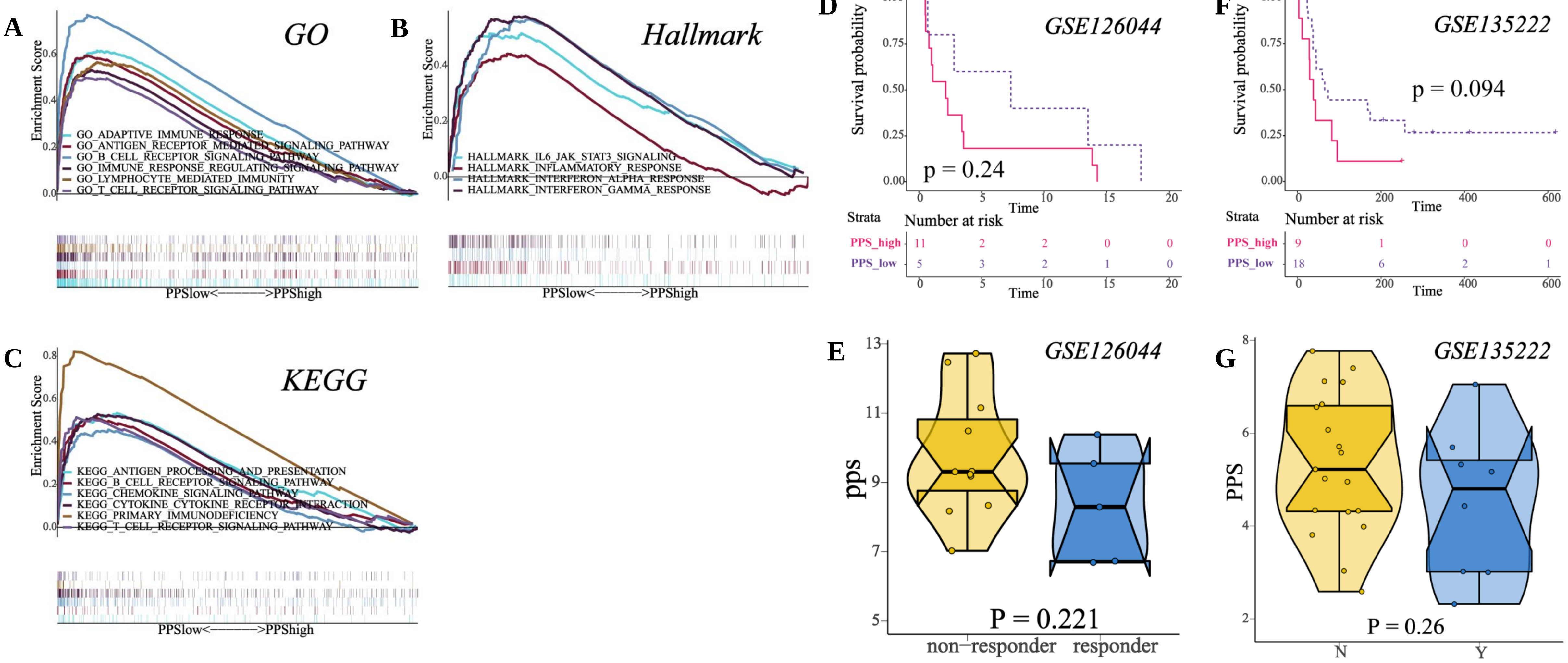

Fig S7. GSEA revealed several important functions or pathways enriched in PPSlow group, and the performance of PPS in two immunotherapy cohort. (A) c5.go.bp.v7.2.symbols.gmt (Gene ontology) was used as reference gene set. (B). h.all.v7.2.symbols.gmt (Hallmarks) was used as reference gene set. (C). c2.cp.kegg.v7.2.symbols.gmt (kegg) was used as reference gene set. several pathways or functions enriched in PPS-low groups were displayed in the graphs (For example, kegg for PPS-low: Antigen processing and presentation, B cell receptor signaling pathway, Chemokine signaling pathway, Cytokine cytokine receptor interaction, Primary immunodeficiency, T cell receptor signaling pathway). (D). Kaplan-Meier curve of patients in PPS-low and PPS-high groups in GSE126044 cohort. Log- rank statistic was conducted to test statistical significance. (E). The distribution of PPS in treatment-responder group and treatment-non-responder group in GSE126044. Kruskal-Wallis statistic was conducted to test statistical significance. (F). KaplanMeier curve of patients in PPS-low and PPS-high groups in GSE135222 cohort. Logrank statistic was conducted to test statistical significance. (G). The distribution of PPS in treatment-responder group and treatment-non-responder group in GSE135222. Kruskal-Wallis statistic was conducted to test statistical significance.

Figure S8

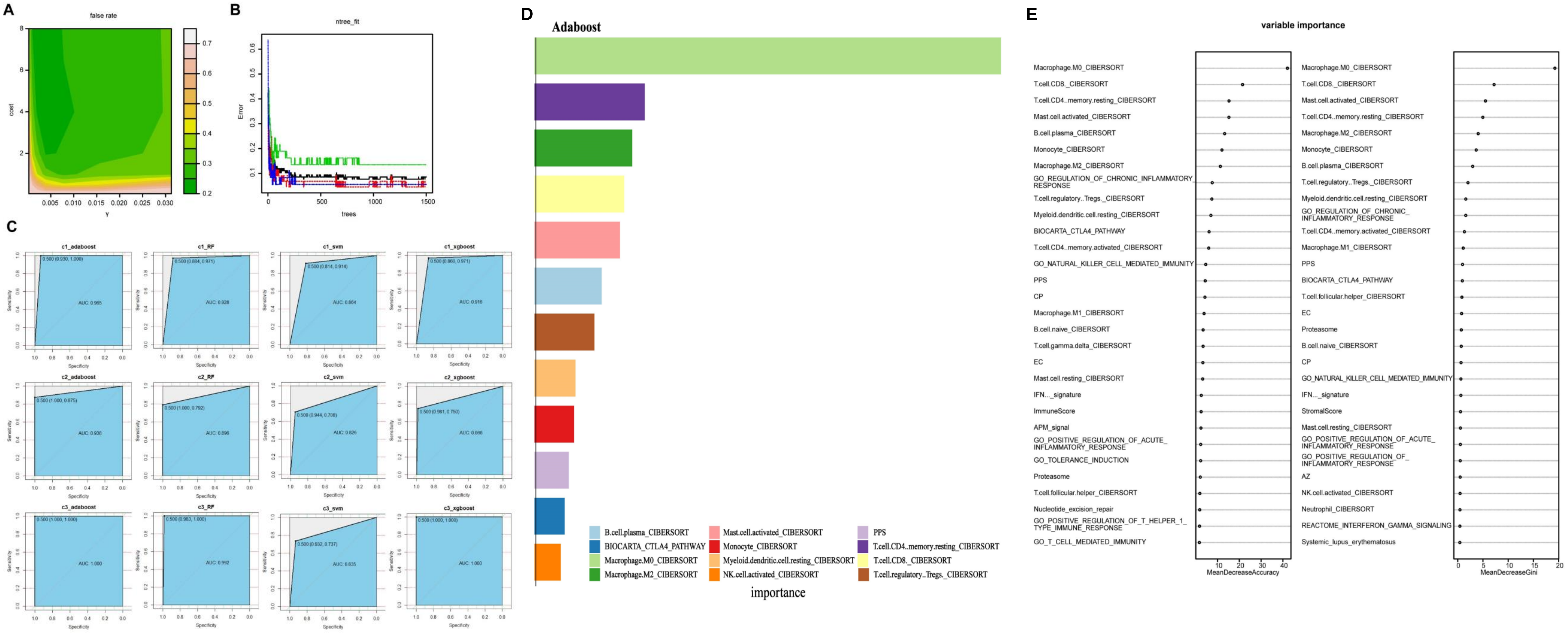

Fig S8. Construction of the classifier. (A). Grid search to find the best gamma and cost values used in the SVM classifier. The darker the color, the lower the false rate. (B). The error plot of random forest model to find the best ntrees parameter. (C). The AUC value for cluster-1, cluster-2 and cluster-3 in different classifiers construted by Adaboost, Randomforest, SVM, and Xgboost. (D). Different colors represent different variables derived from Adaboost. The longer the histogram, the higher is the importance of the variable. (E). The variables importance were ranked by the MeanDecreaseAccuracy or MeanDecreaseGini. Macrophage seems to be the most important variable in the Randomforest model.

Figure S9

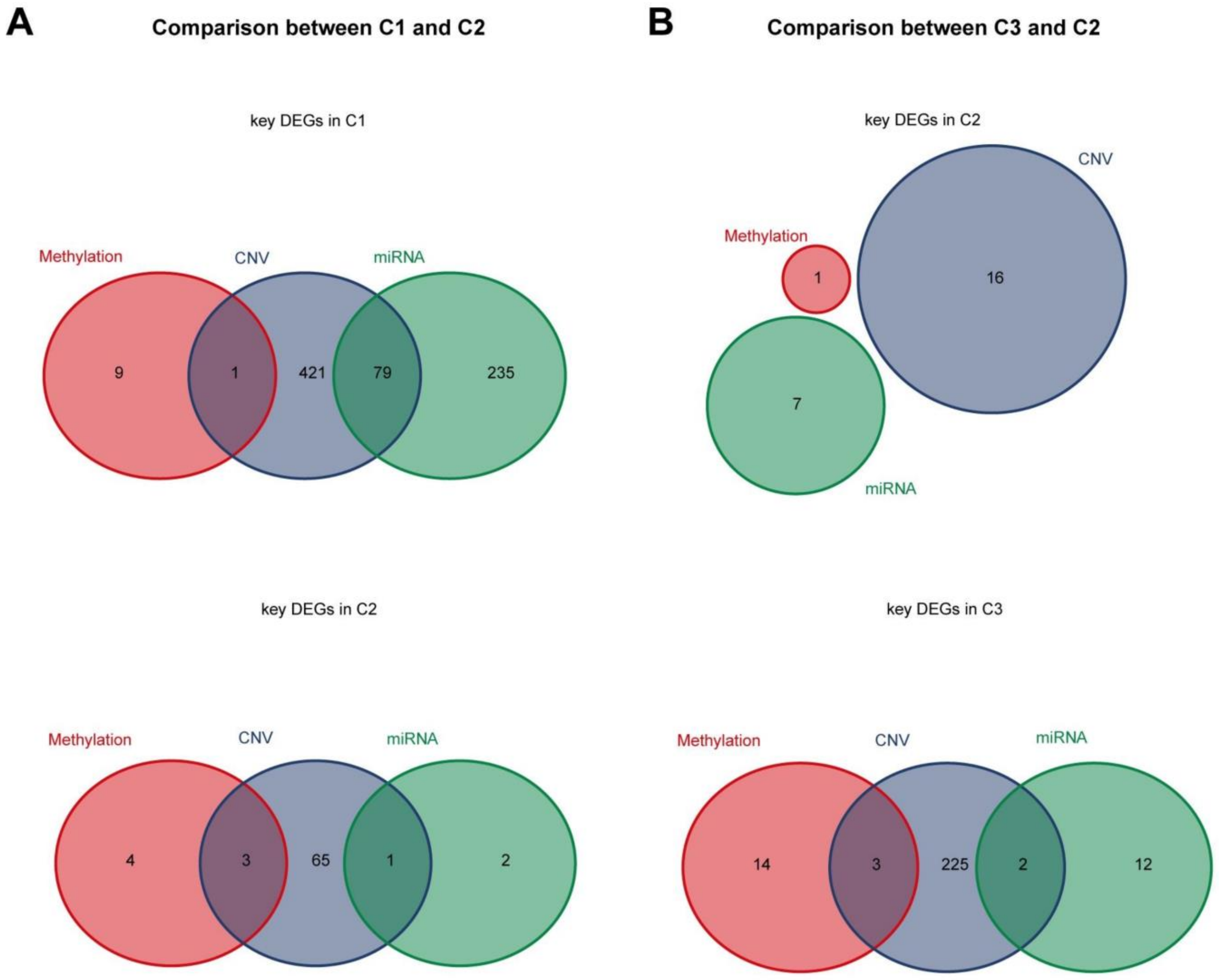

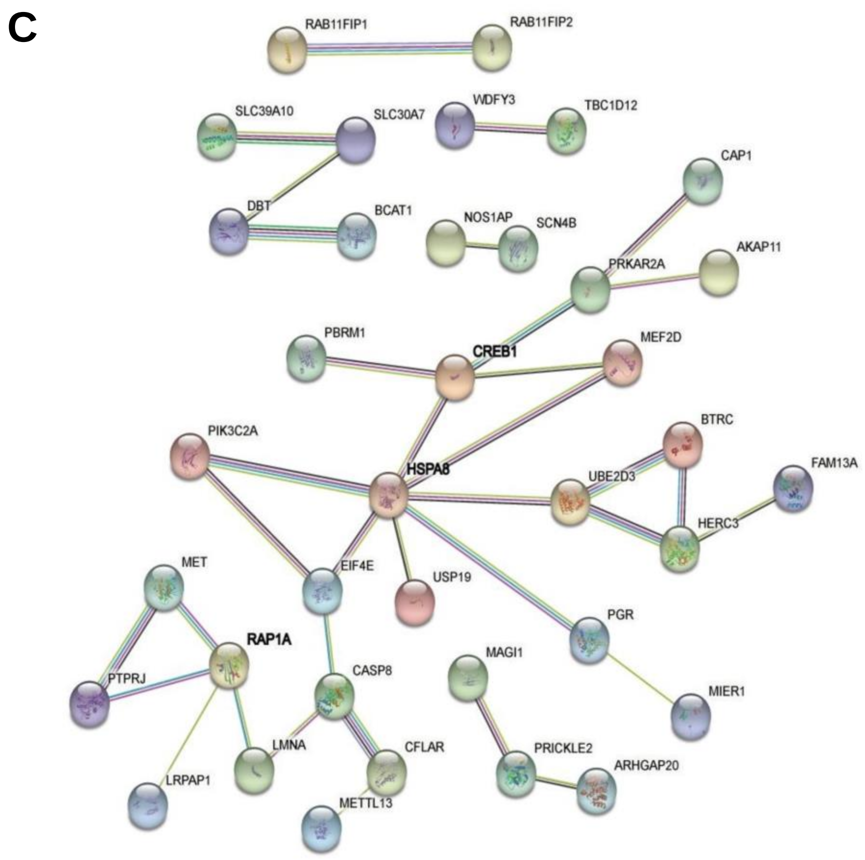

Fig S9. Identification of key DEGs. (A). Venn diagram showed the amount of DEGs upregulated in C1 (up) or C2 (bottom) affected by methylation, CNV, miRNA events. The overlapping part of the venn diagram showed the amount of key DEGs identified in the comparison between cluster-1 and cluster-2. (B). Venn diagram showed the amount of DEGs upregulated in C2 (up) or C3 (bottom) affected by methylation, CNV, miRNA events. The overlapping part of the venn diagram showed the amount of key DEGs identified in the comparison between cluster-2 and cluster-3. (C). Protein-Protein interaction network for key nodes identified in the comparison between cluster-1 and cluster-2. The nodes with four or more connections were shown in bold (including CREB1, HSPA8, RAP1A).

Figure S10

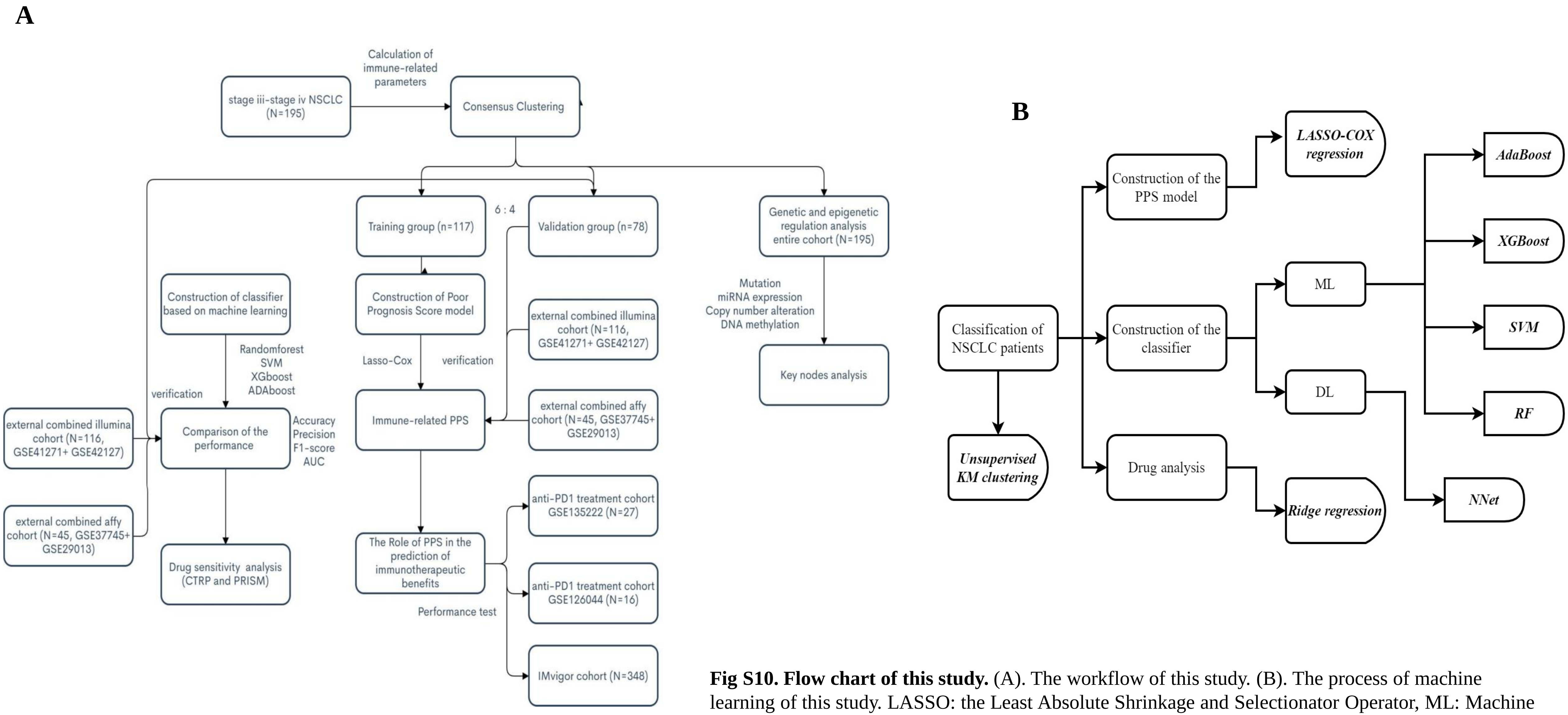

Fig S10. Flow chart of this study. (A). The workflow of this study. (B). The process of machine learning of this study. LASSO: the Least Absolute Shrinkage and Selectionator Operator, ML: Machine Learning, DL: Deep Learning, SVM: Support Vector Machine, RF: Random Forest, NNET: neural network.
