# Supplementary material for: Machine learning and bioinformatics analysis revealed classification and potential treatment strategy in stage 3–4 NSCLC patients
Source: BMC Med Genomics. 2022 Feb 22;15:33. doi: 10.1186/s12920-022-01184-1 (PMC8862473; doi:10.1186/s12920-022-01184-1)
Supplement: Supplementary file 2 — Additional file 2. Supplementary Tables (S1-6). [file 12920_2022_1184_MOESM2_ESM.pdf]

Table S1. Clinical information of patients in affy-combined cohort.

**Characteristics (N=45)**		**No. cases**
Age	age<=65	28
	age>65	17
Gender	female	16
	male	29
Stage	stage iiia	25
	stage iiib	16
	stage iv	4
Type	LUAD	25
	LUSC	20

Table S2. Clinical information of patients in illumina-combined cohort.

**Characteristics (N=116)**		**No. cases**
Age	age<=65	55
	age>65	61
Gender	female	43
	male	73
Stage	stage iiia	61
	stage iiib	50
	stage iv	5
Type	LUAD	74
	LUSC	42

Table S3. Clinical information of patients in two anti-PD1 treatment cohorts.

Cohorts (N=43)		No. cases
GSE135222 (n=27)	responder	8
	non-responder	19
GSE126044 (n=16)	responder	5
	non-responder	11

Table S4. Clinical information of patients in IMvigor cohort.

**Characteristics (N=348)**		**No. cases**
Best.Response	CR	25
	PR	43
	SD	63
	PD	167
	NA	50
Binary.Response	CR/PR	68
	SD/PD	230
	NA	50
Immune.phenotype	desert	76
	excluded	134
	inflamed	74
	NA	64
Gender	female	76
	male	272

Table S5. Correlation between five type of TIICs calculated by CIBERSORT and other algorithms

CIBERSORT_RELATIVE	CIBERSORT_ABS	TIMER	XCELL	EPIC	MCPCOUNTER
0.91	**CD8.T cell**	0.67	0.80	0.71	0.65
0.87	**M1.Macrophage**	0.02	0.50	0.32	0.66
0.80	**M2.Macrophage**	0.58	0.53	0.48	0.49
0.93	**DC.resting**	0.40	0.65	NULL	0.71
0.96	**Neutrophils**	0.42	0.79	NULL	0.49

Table S6. Chi-square test of clinical features among different clusters.

Characteristics		C1	C2	C3	χ^2 C1 vs C2	Pvalue	χ^2 C3 vs C2	Pvalue
**Age**	age<=65	33	31	25	0.800	0.371	0.088	0.766
	Age>65	46	30	29				
**Pathologic_M**	M0	46	48	37	2.167	0.141	<0.001	1.000
	M1	17	8	6				
**Pathologic_N**	N0	10	12	8	0.652	0.419	0.275	0.600
	Ni	65	48	47				
**Gender**	female	31	24	22	0.000	1.000	0.000	1.000
	male	48	37	33				
**Stage**	stage iii	61	53	49	1.537	0.215	0.006	0.937
	stage iv	18	8	6				
